# Bidirectional Promoters as Important Drivers for the Emergence of Species-Specific Transcripts

**DOI:** 10.1371/journal.pone.0057323

**Published:** 2013-02-27

**Authors:** Valer Gotea, Hanna M. Petrykowska, Laura Elnitski

**Affiliations:** DIR/GTB Genomic Functional Analysis Section, National Human Genome Research Institute, National Institutes of Health, Bethesda, Maryland, United States of America; Oxford Brookes University, United Kingdom

## Abstract

The diversification of gene functions has been largely attributed to the process of gene duplication. Novel examples of genes originating from previously untranscribed regions have been recently described without regard to a unifying functional mechanism for their emergence. Here we propose a model mechanism that could generate a large number of lineage-specific novel transcripts in vertebrates through the activation of bidirectional transcription from unidirectional promoters. We examined this model *in silico* using human transcriptomic and genomic data and identified evidence consistent with the emergence of more than 1,000 primate-specific transcripts. These are transcripts with low coding potential and virtually no functional annotation. They initiate at less than 1 kb upstream of an oppositely transcribed conserved protein coding gene, in agreement with the generally accepted definition of bidirectional promoters. We found that the genomic regions upstream of ancestral promoters, where the novel transcripts in our dataset reside, are characterized by preferential accumulation of transposable elements. This enhances the sequence diversity of regions located upstream of ancestral promoters, further highlighting their evolutionary importance for the emergence of transcriptional novelties. By applying a newly developed test for positive selection to transposable element-derived fragments in our set of novel transcripts, we found evidence of adaptive evolution in the human lineage in nearly 3% of the novel transcripts in our dataset. These findings indicate that at least some novel transcripts could become functionally relevant, and thus highlight the evolutionary importance of promoters, through their capacity for bidirectional transcription, for the emergence of novel genes.

## Introduction

The question of how new genes and new functions originate remains one of the most intriguing open questions in evolutionary genetics [1]. For example, the duplication of existing genetic material was proposed as a solution to this problem nearly 100 years ago [2], [3]. Since then, the duplication model has provided the basis for explaining the expansion of genomes and diversification of various protein families [4], [5], [6], [7], becoming widely popular with the publication of Ohno’s “Evolution by Gene Duplication” [8]. The model proposes that the presence of two copies of the same gene frees one of them from functional constraints, allowing the second copy to mutate and develop new functions. The role of gene duplication in evolution is indirectly emphasized later on by François Jacob, who dismissed as a possibility the idea that functional proteins emerge through the random association of amino acids [9]. It follows therefore that the gene duplication is the most widely accepted mechanism for the diversification of the gene repertoire of every species.

In addition to the mechanism of gene duplication, recent evidence accumulates to support the diversification of the gene repertoire through the emergence of novel genes from previously untranscribed regions. Examples of newly emerged lineage-specific genes include our previous work in cow [10], and extend to organisms as diverse as *Plasmodium vivax* [11], yeast [12], [13], Drosophila [14], [15], [16], and primates [17], [18], [19]. Nonetheless, Kaessmann recently highlighted the lack of a specific mechanism to account for the emergence of new genes from genomic regions lacking prior gene functions, i.e. “from scratch” [20]. Here we propose and provide evidence to support such a mechanism for the emergence of novel genes. Specifically, we propose that some functional promoters initiate and establish transcription opposite to an endogenously controlled gene. If such regions lacked previous transcription activity, the activation of bidirectional transcription leads to the emergence of novel, lineage-specific transcripts. This mechanism is based on previous, limited associations between bidirectional promoters (BDPs) and lineage-specific transcripts in mammals [10]. Given the propensity of promoters for bidirectional transcription [21], [22], [23], we estimate that the lineage-specific activation of BDPs should be an important mechanism for the emergence of novel transcripts. Such transcripts would therefore emerge from regions without prior genic function, and would be spliced based on the presence of appropriate resident sequences that provide necessary spicing signals. Such novel transcripts provide a molecular pool for functional diversification and adaptive change.

## Results

### Novel Transcriptional Units in the Human Genome

The proposed evolutionary mechanism predicts the presence of lineage-specific BDPs and associated lineage-specific novel transcripts in any species. BDPs are defined as regions flanked by two head-to-head (i.e. antisense-oriented) transcripts separated by at most 1 kb [24], while lineage-specific BDPs could be defined as those where one transcript is conserved across multiple species and the other is specific to a single lineage, such as the case of the BDP flanked by the *CYB5R4* gene and its DV834581 partner transcript in cow [10]. Here we investigated the impact of this mechanism on the human transcriptome through finding primate-specific BDPs, because human is the species that presents both the deepest transcriptome data, necessary to capture non-conserved transcripts, and the highest relevance for the biomedical scientific community. To identify lineage-specific transcripts emerged through the activation of bidirectional transcription from active promoters, we started with identifying BDPs using transcripts annotated in the RefSeq, UCSC KnownGene and spliced EST reference sets (see Methods), and avoiding short-lived RNA-molecules [22], [23]. We limited our initial set to 1,945 BDPs with one transcript lacking an annotated open reading frame (ORF) and the other transcript corresponding to an annotated protein-coding gene. The lack of annotated ORF is consistent with non-coding expectations for most novel transcripts, while the protein-coding gene facilitates the evaluation of the evolutionary conservation and orthology assignment between genomes. At the same time, this makes for a conservative dataset because it ignores potential rare cases where novel transcripts have annotated ORFs, or cases where novel transcripts emerge from promoters of genes that do not code for proteins. If more than one transcript pair flanked a BDP region, we retained the pair with the closest transcription start sites (TSS), from which we further selected 1,467 pairs where the protein-coding transcript was conserved in mouse (see Methods), which we refer to as “anchors”. We further eliminated 400 pairs where we found evidence of transcription at the mouse locus orthologous to the non-coding transcript, and we used these as a reference set for conserved non-coding transcripts (cncRNAs).

Our final data set consisted of 1,067 BDPs flanked by anchor transcripts on one side, and transcripts considered to be primate-specific transcripts on the other, referred to as “promoter-identified novel transcripts” (PINTs). To ensure that PINTs were valid transcription units controlled by BDPs, we had their transcriptional activity experimentally tested within the context of the GENCODE framework [25] in eight tissues (brain, heart, kidney, liver, lung, muscle, spleen, testis). In 34 of 39 cases examined, both the PINT and corresponding anchor were transcribed in at least one of the eight tissues (Table S1).

It has been shown that BDPs flanked by two protein coding genes are randomly distributed across the genome, as their counts are significantly correlated with the gene abundance in each chromosome [26]. We therefore expected that PINTs also display random genomic distribution, and compared their chromosomal distribution to that of protein coding genes with potential for initiating transcription upstream from their own promoter (see Methods). We found no significant difference between the two sets (Fig. S1), consistent with the BDP random distribution. Furthermore, to assess whether PINTs emerge due to specific functional properties of the anchor genes, we tested for gene ontology (GO) enrichment among anchor genes (see Supplementary Methods). No functional category showed a significant enrichment, indicating that the emergence of PINTs occurs randomly across the genome and is consistent with a model that is not reliant on specific properties of genes defined as anchors.

### PINT-anchor Expression Correlation

Identification of PINTs was based on structural characteristics of BDPs, but our evolutionary model, which involves the activation of bidirectional transcription, implies that the regions separating PINTs and their anchors also share functional characteristics of BDPs. Specifically, Trinklein *et al.* have shown that expression of genes flanking BDPs is significantly correlated [27], which could therefore be also expected for PINTs and their anchors. To assess whether PINTs and anchors also present a correlated pattern of expression, we created a relative expression difference (RED) parameter that measures the difference between expression levels across several tissues for two transcripts (see Methods). The parameter can capture the known correlation between the expression of protein-coding genes flanking BDPs (Fig. S2), with lower RED values indicating higher expression correlation. To evaluate expression levels, we used data from the Affymetrix Human Exon 1.0 ST microarrays [28]. Specifically, we used expression values associated with probes mapping in exons closest to promoters to avoid additional signals from hybridization of transcripts from alternative promoters not relevant to the activity of BDPs (as we showed in [29]).

**Figure 1:**
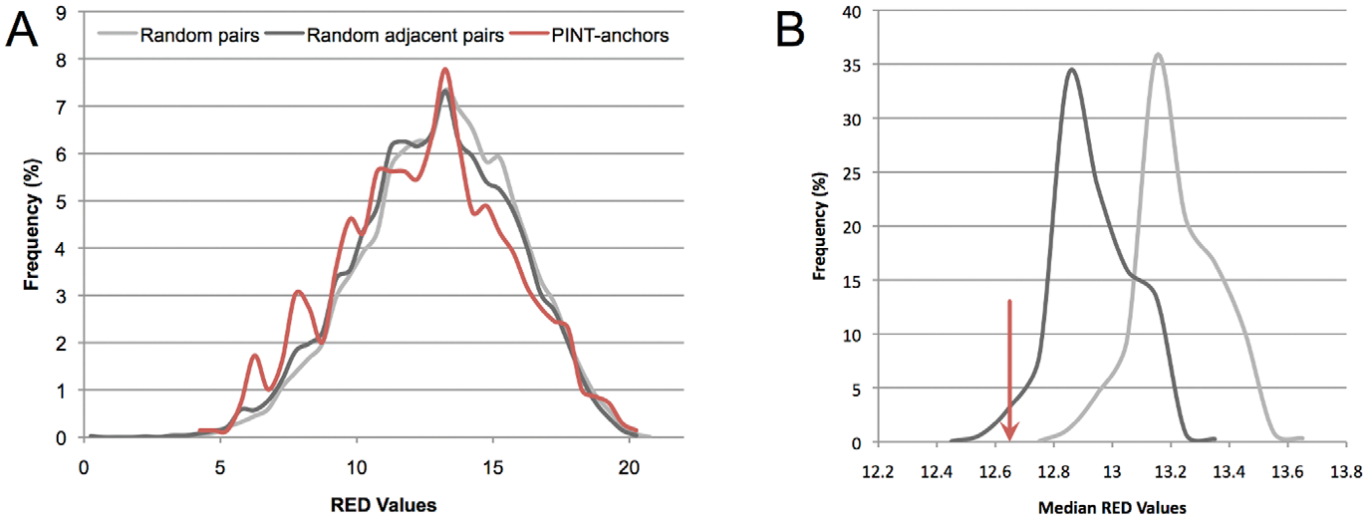
The expression of PINTs is correlated with the expression of anchors. (**A**) RED values associated with PINT-anchor pairs (shown in red, median value 12.65) are significantly lower than values obtained for pairs of randomly chosen coding and non-coding transcripts (light-gray distribution; *P* = 7.6×10^−7^, MWU test) and values obtained for randomly chosen pairs of adjacent coding and non-coding transcripts (dark-gray distribution; *P* = 0.0026). Random distributions consist of 1,041,000 RED values (1,500 replicates of 694 pairs). (**B**) The same comparison is illustrated by comparing the medians of distributions. The median RED value for PINT-anchor pairs (red arrow) is significantly smaller than the expected value based on pairs of randomly selected coding and non-coding transcripts (light-gray, 1,500 values, *P*<6.7×10^−4^) and random pairs of adjacent coding and non-coding transcripts (dark grey, *P* = 0.035).

The distribution of RED values for PINT-anchor pairs has a median value of 12.6 (Fig. 1). Using a one-sided Mann Whitney U test (MWU), we found these values to be significantly lower than RED values computed for randomly associated protein-coding and non-coding genes (median 13.2, *P* = 7.6×10^−7^). We also found them to be significantly lower than RED values computed for adjacent pairs of protein-coding and non-coding genes, but which are not controlled by BDPs (see Methods, Fig. 1; *P* = 2.6×10^−3^, MWU test). These data indicate that regions separating PINTs from their anchors share not only structural, but also functional similarities with BDPs, supporting the model of PINT emergence through the conversion of unidirectional into bidirectional promoters.

### PINTs are Poorly Conserved Across Vertebrates

**Figure 2:**
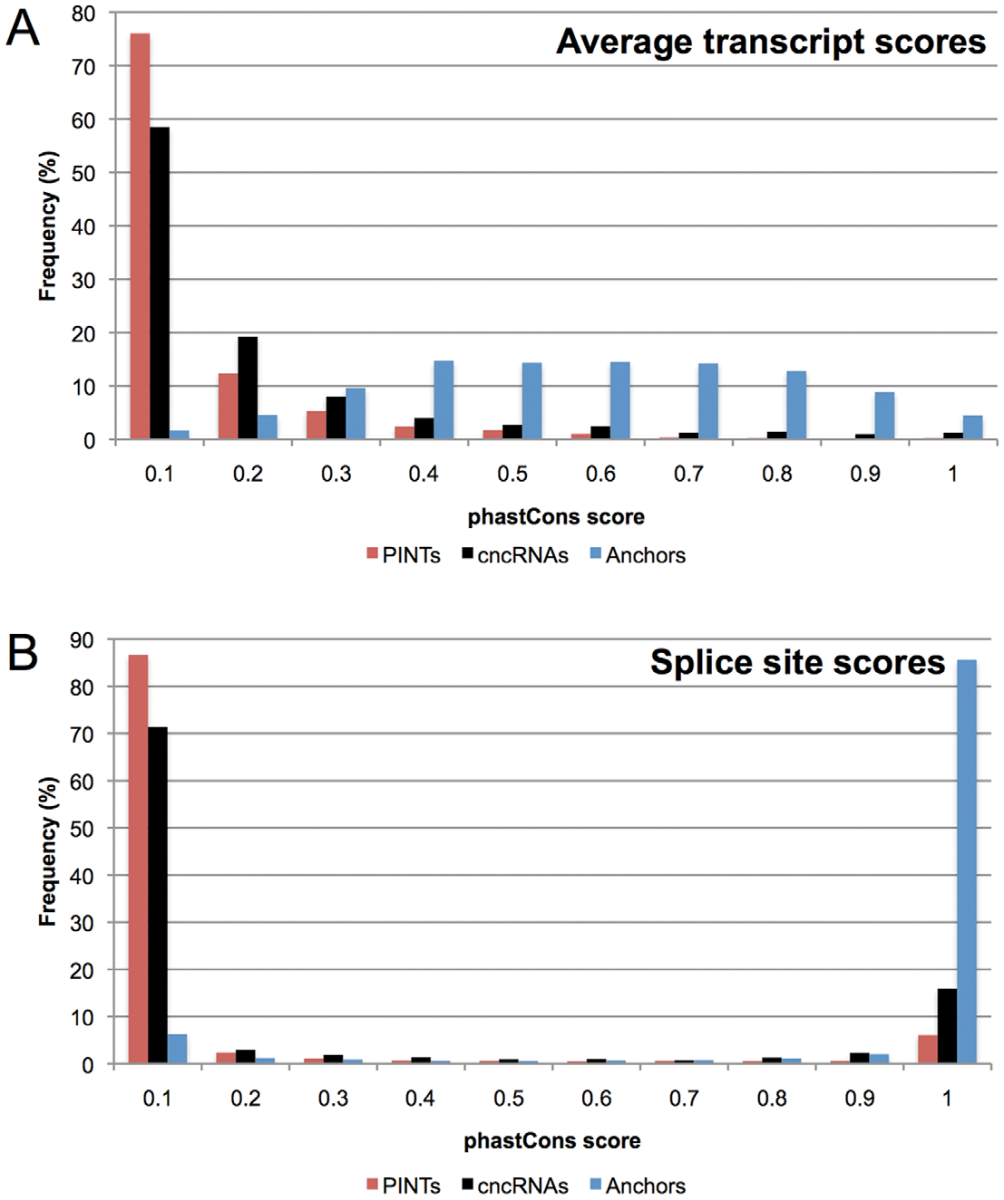
Low conservation of PINTs illustrated by phastCons scores. (**A**) Distribution of transcript-wide phastCons average-scores for PINTs (red, median 0.026), cncRNAs (black, median 0.06), and anchors (blue, median 0.535). (**B**) Distribution of phastCons scores for all splice sites for PINTs, cncRNAs, and anchors (medians of 5×10−4, 0.0025, and 0.9995, respectively).

Our method of identifying PINT candidates is intended to find transcripts that have emerged in the primate lineage. Nevertheless, by using only mouse as a reference species (see Methods), some of the identified candidates may represent transcripts that were specifically lost in the mouse or rodent lineage. Ideally, the latter alternative could be ruled out by analyzing orthologous loci in other placental non-primate species, but suitable extensive transcriptome datasets do not currently exist, despite recent advances in the field [30]. The former alternative could be supported by low conservation of PINT sequences, since it would be expected that recently emerged transcripts exhibit lower sequence conservation levels across vertebrates relative to levels observed for ancestral transcripts. We therefore compared the sequence conservation in the exons of PINTs to levels observed in exons of cncRNAs, which represent comparable non-coding transcripts but which we found to be conserved between human and mouse. Using phastCons scores (computed from 17-way alignment of vertebrate species, see Methods) as a measure of conservation, we found that PINTs exhibited significantly lower conservation than cncRNAs (Fig. 2A; median phastCons scores 0.026 and 0.06, respectively; *P* = 4.9×10^−11^, MWU test). The difference remains significant even after removing annotated repeats (median phastCons scores of 0.026 and 0.074, respectively; *P* = 1.3×10^−11^, MWU test). As expected, PINTs were also significantly less conserved than anchor genes (median phastCons score 0.535; *P* = 1.2×10^−299^, MWU test). These data indicate that the conservation of PINTs is more consistent with their being novel transcripts rather than ancestral transcripts depleted in rodents.

To account for the possibility that PINTs represent RNA molecules with conserved secondary structures but lower nucleotide conservation due to compensatory mutations, we also investigated conservation at the level of consensus splice dinucleotides. These sites are known to be evolving under purifying selection, with newly acquired splice sites (SS), such as those of alternatively-spliced exons, being subject to relaxed constraints [31]. We found phastCons scores to be the lowest for PINT SSs (median score of 5×10^−4^, Fig. 2B), and significantly higher for SSs used by both cncRNAs (median 0.0025, *P* = 1.5×10^−37^, MWU test) and anchors (median 0.9995, *P* = 0, MWU test). This finding reinforces the hypothesis that PINTs represent transcripts with characteristics of newly-emerged, primate-specific transcripts.

### Splice Site Recruitment

Additional support for the novel character of PINTs could be provided by their selective use of splice sites (SSs). Newly emerged transcripts should lack any type of constraints, and therefore the splicing of such molecules into mature RNA products should utilize any splice signal (defined here as “GT” and “AG” dinucleotides on the transcribed strand) that meets the requirements of the splicing process, such as reasonable maximum entropy scores [32] and required neighboring sequences [33]. With the use of an appropriate marker, the use of SSs could be tested against the available splice signals across the transcripts’ genomic loci. A suitable marker for this test could be considered the origin of splice signals in transposable elements (TEs). The reasons are twofold. Not only are TEs known to carry ready-to-use splice signals, but such signals are rarely adopted into functional transcripts because of the risk of resulting disease phenotypes [34].

**Figure 3:**
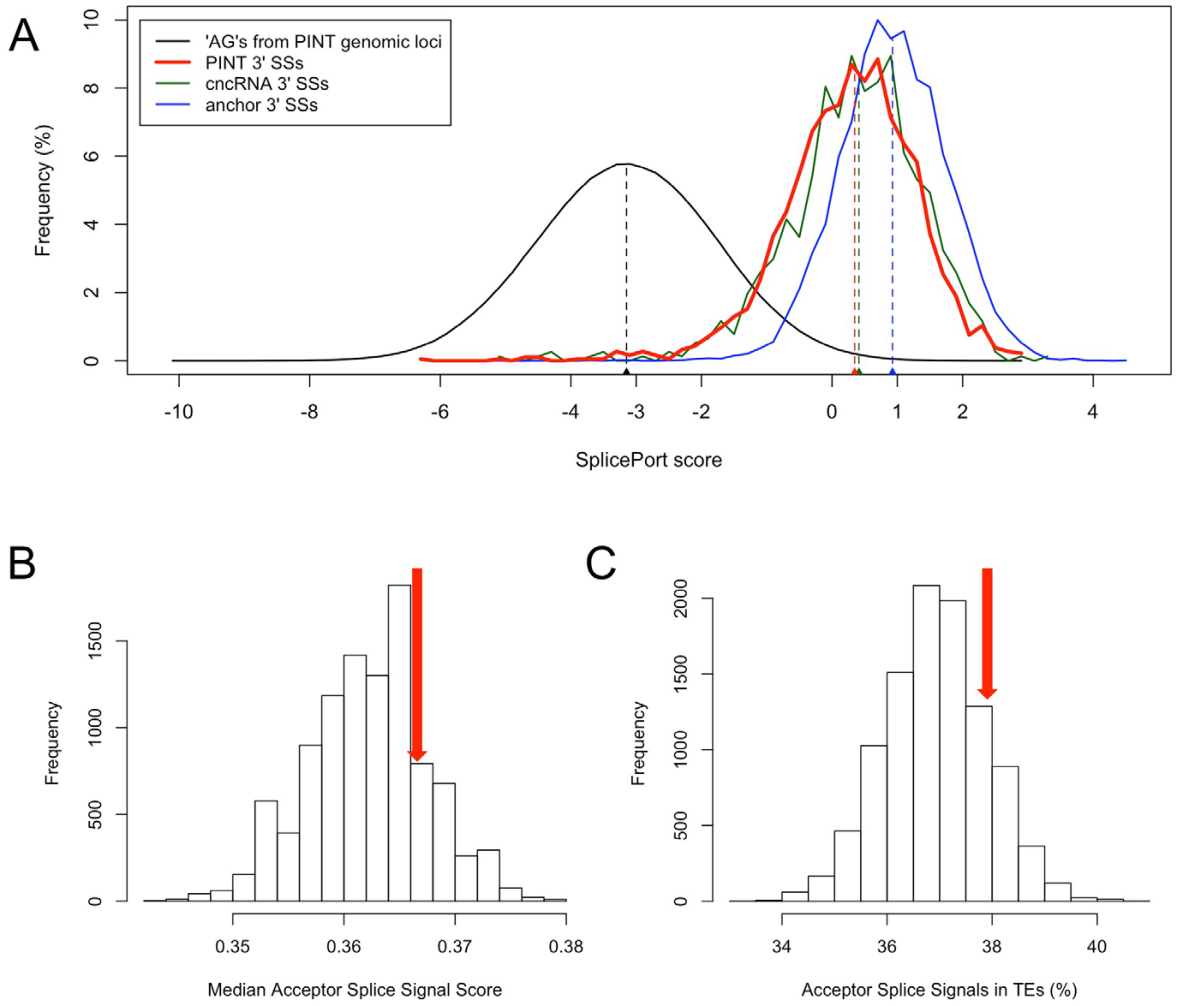
Assesing the strength and TE origin of SSs. (**A**) The acceptor (3′) SSs used by PINTs exhibit higher SplicePort scores (red; median 0.37) than background splice signals (black; median −3.15, *P* = 0, MWU test), but lower than score of SSs used by cncRNAs (green; median 0.41, *P* = 0.07) and anchors (blue; median 0.96, *P* = 4.5×10^−148^). Dashed vertical lines indicate median values of corresponding distributions. (**B**) Random sets of 1,939 “AG” dinucleotides (10,000 replicates) were selected to match the score distribution of acceptor SSs used by PINTs to determine the expected fraction of TE-derived SSs. The confirmation of the score distribution fit is provided by the non-significant difference between the median of PINT acceptor SS scores (0.37) and random samples (*P* = 0.19). (**C**) Using the same sets as in (B), we show that the fraction of TE-derived acceptor SSs in PINTs (37.9%, red arrow) is not significantly different (*P* = 0.16) from expectation.

By analyzing the TE origin of SSs utilized by different transcripts, we found that PINTs had the highest fraction of TE-derived SSs: 37.9% and 23.4% for acceptor (3′) and donor (5′) SSs, respectively. The corresponding fractions were significantly lower for both cncRNAs (acceptor: 29.8%, *P* = 3.9×10^−5^, Fisher’s exact test; donor: 15.7%, *P* = 4.5×10^−6^) and anchors (acceptor: 0.7%, *P*<10^−16^; donor: 0.6%, *P*<10^−16^). We compared these values to background expectations based on available signals available at the genomic loci (after controlling for signal strength, see Methods), and found that TE-derived acceptor SS usage in PINTs was not significantly different from the expected value. Specifically, the 37.9% fraction of TE-derived acceptor SSs used by PINTs was not significantly different than the expected 36.5% (*P* = 0.16, Fig. 3), in agreement with random SS usage and relaxed or absent selection pressure acting on PINTs. In contrast, the values observed for cncRNAs and anchors were both lower than expectations (35.5% and 9.6%, respectively, *P*<10^−4^ in both cases; Fig. S3), indicating that purifying selection is acting on SSs of older transcripts. In the case of donor SSs, all observed values were significantly lower than expectations (data not shown), suggesting that donor SSs are subject to additional constraints not captured by our model. Nevertheless, the randomness of acceptor SS usage in PINTs strongly agrees with a recent timeframe for the emergence of PINTs, precluding the selective constraint seen for the SSs of older and conserved transcripts.

### Preferential TE Accumulation Upstream of Promoters

**Figure 4:**
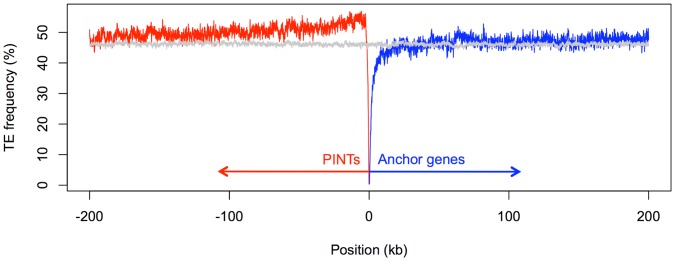
TEs accumulate preferentially in regions upstream of promoters. The frequencies of TE-derived sequences are shown in a 400-kb window centered on the TSS of anchor genes: PINT and anchor loci are plotted in red and blue, respectively, while the genome-wide average is shown in gray (10,000 randomly chosen 400-kb intervals).

The lower conservation of PINTs and their random SS usage prompted us to investigate the impact of TEs on PINT sequences. Consistent with our previous observations, we found annotated TEs in the exons of significantly more PINTs (76.4%) than of either cncRNAs (62%, *P* = 4.9×10^−8^, Fisher’s exact test) or anchors (34.6%, *P* = 10^−86^). The big difference between the expected fractions of TE-derived signals at PINT and anchor loci (36.5% and 9.6%, respectively; see above) suggested that TEs occur at different rates across the corresponding genomic loci. Indeed, we found that PINT loci contain significantly more TEs than anchor loci (median values of 46.9% *vs*. 35.3%, *P* = 1.9×10^−31^, MWU test). In a plot of TE frequency in a 400-kb window centered on the anchor transcription start sites (TSS) the difference in TE content is highlighted by a peak located in the genomic loci of PINTs, upstream from anchor loci (Fig. 4). The peak in TE frequency is consistent with relaxed functional constraints at PINT as compared to anchor loci. It follows that relaxed functional constraints, and consequently increased TE occurrence, should also be characteristic to regions located downstream from anchor loci. However, we found no perceivable increase in TE frequency downstream from anchor loci (Fig. S4), which suggests that the preferential TE accumulation upstream of their promoters is likely favored by properties specific to active regulatory regions such as targeted histone modifications and associated open chromatin.

The co-occurrence of high TE frequency and PINTs makes it tempting to speculate that increased TE activity is linked to the emergence of PINTs. If this were the case, we expect no peak of increased TE frequency when protein-coding genes not associated with PINTs are interrogated. However, when analyzing the entire set of protein coding genes, we found that preferential TE accumulation upstream of gene loci is a characteristic shared by many genes, which we confirmed in the genomes of human (Fig. S5) and mouse (Fig. S6), as well as in non-mammalian species such as chicken (Fig. S7). The phenomenon is emphasized when only lineage-specific TEs (e.g. most recently active) are investigated, such as human Alu (Fig. S8), mouse B1 (Fig. S9), and chicken CR1 repeats (Fig. S10). These findings indicate that the effect is not specifically associated with any species or TE type, but is likely a consequence of properties of regulatory DNA in promoter regions.

### Positive Selection Acting on PINTs

One of the most important evolutionary questions related to the emergence of PINTs in any genome is whether such novel transcripts could acquire novel functions. Owing to their young age, we postulate that most PINTs have a neutral functional impact on the host organism. Nonetheless, some of these transcripts could acquire novel functions, and consequently be subjected to positive selection. Finding such transcripts could help with the understanding of lineage-defining traits, and with the prioritization of candidates for experimental validation.

In the case of protein coding genes, a classic sign of positive selection is a high ratio of non-synonymous to synonymous substitutions (dN/dS). Since PINTs lack conventional ORFs and have low coding potential (Fig. S11, S12), we searched for evidence of elevated rates of lineage-specific substitutions, which is as an indicator of positive selection for non-coding sequences [35]. The high TE content of PINTs makes it feasible to propose a novel test for detecting signs of positive selection in PINT TE-derived fragments. The power of this test relies on the numerous specific TE homolog fragments located in intergenic regions that can be used to derive empirical distributions of expected human-specific substitution rates for each TE fragment. The set of specific homologs exhibits no sequence bias, evolves in a mostly neutral environment, and is rarely affected by transcription-coupled repair that might artificially lower the expected rate of substitutions [36]. Local substitution rates can also be incorporated into the model to account for the mutagenic effect of increased recombination favored by certain TEs [37]. Moreover, to rule out GC-biased gene conversion [38] as an alternative explanation to accelerated lineage-specific substitution rates, we searched for mutational hotspots that favor strong (G or C) over weak (A or T) nucleotides (see Supplementary Materials).

We applied this test to all TE-derived fragments longer than 50 bps that are embedded in PINTs and have human-specific substitution rates of at least 2% (see Methods). While this threshold is empirically chosen, it characterizes many of the human accelerated regions identified by Pollard et al. [39], and serves to minimize the rate of false positives and to increase the power of detection after correction for multiple testing. In total we found 51 such fragments, of which 31 had significantly high rates at the individual 5% error level and overall 10% FDR (see Methods). Three of these fragments were located in mutational hotspots resembling regions of GC-biased gene conversion (Table S2, Fig. S13, S14). The remaining 28 regions indicate that the corresponding PINTs have been subject to positive selection in the human lineage.

### Functional Impact of Adaptive Mutations

**Figure 5:**
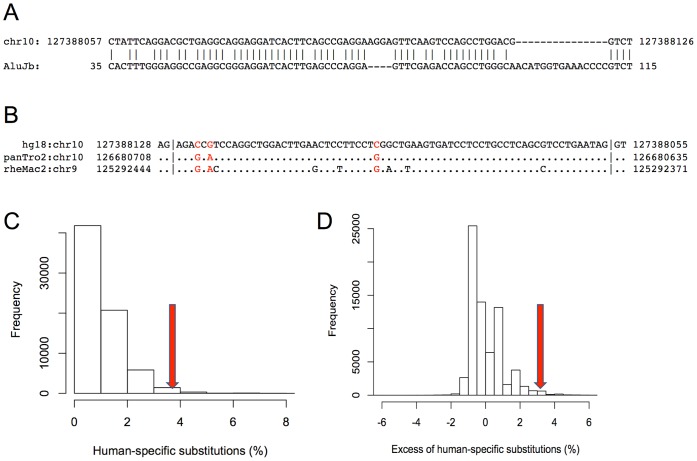
Assessing positive selection in the third exon of the AK094354. (**A**) Alignment of the 70-bp human exon to the AluJb consensus sequence identifies positions 35–115 in the AluJb consensus as the source of the exon. (**B**) Alignment of the AluJb-derived exon between human, chimp and macaque orthologs. Positions of human specific changes are shown in red, and vertical bars separate the adjacent flanking consensus splicing dinucleotides. (**C**) Distribution of human-specific changes in 70,275 AluJb intergenic homologs that correspond to the 35–115 AluJb consensus region. (**D**) To take local mutation bias into account, the values are expressed as excess over local human-specific substitution rates (right panel; see Methods). Red arrows correspond to values observed for the 70-bp exon of interest, and represent significantly high substitution rates (*P* = 0.006 and *P* = 0.004, respectively) in both cases.

We used data on positive selection to prioritize PINTs for functional testing. The top ranking TE fragment in terms of its significance (Table S2) is a 70-bp fragment corresponding to coordinates 35–115 in the AluJb consensus sequence (Fig. 5A). It spans the entire length of the third exon of a non-coding transcript AK094354 and contains three (4.28%) human-specific substitutions (Fig. 5B), which yield a highly significant P-value both before and after correction for local mutation bias (Fig. 5C).

**Figure 6:**
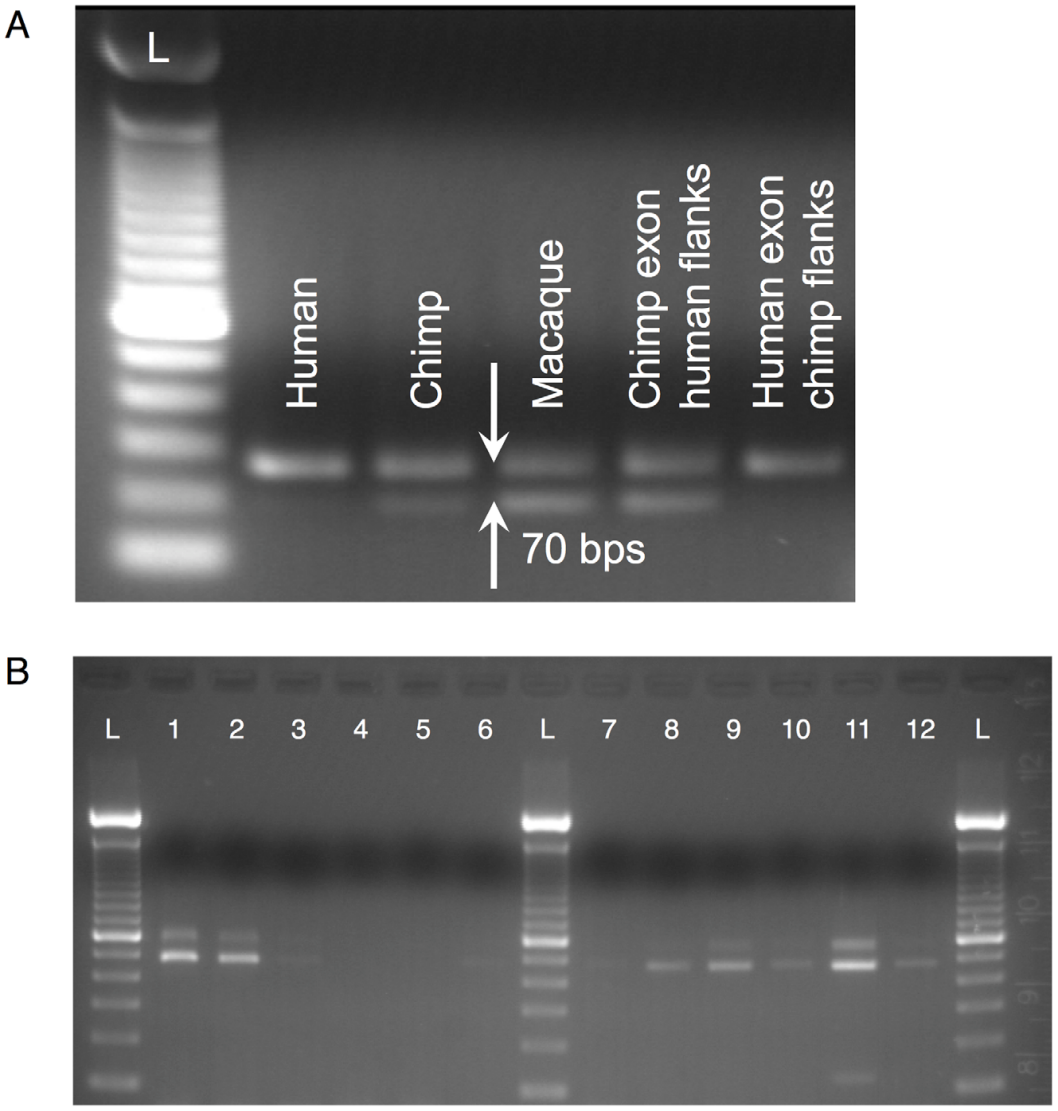
Experimental evidence for the splicing of the AluJb-derived exon 3 in AK094354. (**A**) The splicing efficiency of the AluJb-derived exon was tested in a minigene splicing assay both with the human sequence, as well as with the chimp and macaque orthologs. The upper band (which includes the 70-bp exon) is the strongest in human both visually and quantitatively (Fig. S15), whereas the lower band corresponds to the exon being skipped. The Invitrogen 100-bp DNA ladder (L) was used as a marker. (**B**) RT-PCR of the transcript in 12 human tissues: 1 - brain, 2 - spleen, 3 - muscle, 4 - placenta, 5 - heart, 6 - liver, 7 - lung, 8 - stomach, 9 - kidney, 10 - intestine, 11 - testis, 12 - colon. Two dominant bands (581 and 466 bps) were found in ten tissues (no product observed in placenta or heart), corresponding to isoforms with alternatively spliced exon 2 of the transcript (115 bps). A shorter band can be observed for testes, which corresponds to non-specific amplification from chromosome 6 (verified by sequencing).

Based on the locations of the substitutions in this noncoding exon we predicted that they could affect the exonic splicing regulatory elements and splice sites [33], [40]. We evaluated the impact of the human-specific substitutions by scoring the donor and acceptor SSs in human, chimp and macaque and found the highest predicted splicing efficiency in human (Table 1). The *in silico* prediction was verified experimentally with a minigene splicing assay (see Supplementary Materials, Table S3), which showed that the exon is spliced much less efficiently from chimp and macaque than from human sequences (Fig. 6A, S15). Furthermore, an *ex vivo* survey of transcriptomes from twelve tissues revealed that the exon is present in all transcript isoforms (Fig. 6B), confirming the predicted high splicing efficiency in human. These data indicate that the exon originating in a primate-specific TE acquired constitutive splicing in human due to the three human-specific mutations.

**Table 1:** SplicePort scores for SSs flanking the third exon of the AK094354 transcript.

Species	Acceptor (3′) SS Score	Percentile	Donor (5′) SS Score	Percentile
Human	0.424794	25.35	0.336637	25.59
Chimp	0.148871	16.04	0.123589	16.88
Macaque	–1.33083	0.27	–0.503675	4.39

Chimp and macaque scores were computed for sequences orthologous to human SS (alignments of the exonic regions are shown in Fig. 5B). The highest values were observed in human, and the lowest in macaque. Score percentiles were computed based on the original set of SSs used for SplicePort training.

## Discussion

Our findings highlight an evolutionary model in which established unidirectional promoters gain the capacity for bidirectional activity to generate stable transcripts that can undergo splicing. This process can be easily generalized to all unidirectional promoters, and is fundamentally different from the process of gene duplication, which usually involves tinkering with copies of existing genes. The emergence of PINTs allows for the creation of transcripts from sequences without prior gene function, which in turn could contribute novel characters during evolution. The mechanism is likely to be present in all genomes owing to the presence of bidirectional promoters (shown here and elsewhere), and should be regarded as an important source of transcriptional innovation, driven by the promoter propensity for antisense transcription [21]. A conservative comparison with other regions (see Supplementary Materials) indicates that novel transcripts emerge upstream of protein-coding genes at a rate nearly five-fold higher than elsewhere (Fig. S16). It is notable that less than 5% of PINTs contain sequences duplicated from other genes, which are located toward the 3′ end of PINTs, not their 5′ ends. This indicates that gene duplication plays an insignificant role in the emergence of PINTs.

While the molecular mechanism for the PINT emergence is beyond the scope of this study, previous studies have shown that bidirectional transcription could emerge as a result of the inactivation of the Ssu-72 protein factor that has been associated with promoting divergent transcription [41]. One could also imagine that activation of bidirectional transcription could involve the suppression of transcriptional silencers or existing boundary elements (e.g. CTCF), or the acquisition of bidirectional regulatory elements such as GABPA [42] and ZNF143 [43]. It is tempting to speculate that at least in some instances such events could be initiated as a result of TE insertions, given the preferential TE accumulation upstream of promoters (Fig. 4). Other mechanisms could include the activation of bidirectional transcription as a result of chromatin alterations following TE insertions. Further studies are necessary to clarify the link between TEs and emergence of PINTs, since we found few PINTs relative to the number of loci where PINTs could have emerged.

The preferential accumulation of TE-derived sequences upstream of active genes (Fig. 4, S5) represents a novel observation in genomes. The evolutionary importance of such insertions is likely to be multifold, such as through enlarging inter-genic distances, diversifying the genomic context from which PINT exons could be derived, interfering with existing regulatory elements or providing new ones. At the same time, TEs could influence the transcription process through their methylation status. For example, highly expressed PINTs are significantly fewer than expected in cerebellum (Fig. S17), where the methylation of Alu elements is high [44]. In contrast, testes is the tissue with the most highly expressed PINTs, where the methylation levels of Alu elements are minimal [45]. Therefore it is possible that the transcription of many PINTs was initiated in testes, consistent with the “out of testes” hypothesis for the emergence of new genes [20].

The evolutionary importance of BDPs in generating novel functions is highlighted by the detection of positive selection in a subset of PINTs, indicating that among the novel transcripts that have emerged from non-genic regions, some could provide features that prove beneficial in the adaptive evolution of a given lineage. It is important to note that we found adaptive mutations that result in both exon gain and loss. In contrast to the case of AK094354, we found another exon under positive selection where the human-specific substitutions are associated with lower splicing efficiency (Table S4). This is a 118-bp exon which is part of the minor non-coding isoform of C22orf45, is derived from an AluSx repeat, and contains three human-specific substitutions (2.5% substitution rate). The exact functional consequence of such tinkering remains to be determined through dedicated experiments.

Together, these data support a model whereby the conversion of unidirectional into bidirectional promoters is an important evolutionary mechanism capable of generating novel transcripts with functional relevance. Such transcripts could gradually acquire diverse functions as RNA molecules equivalent to lncRNAs, which are involved in transcriptional and epigenetic gene regulation [46], [47], [48], or the capacity to encode proteins [19]. This evolutionary model could explain why protein-coding genes derived from TEs are enriched among the genes controlled by BDPs [49]. Therefore, bidirectional promoters and associated novel transcripts should be regarded as important contributors to the pool of lineage-defining characters in the evolution of genomes.

## Materials and Methods

### Transcript Data

For human, we used the refFlat, knownGene, and intronEst transcript annotations from the hg18 UCSC Table Browser. To define protein-coding loci, we selected transcripts from the RefSeq and knownGene sets that have properly annotated ORFs, and combined into the same locus all transcripts that share at least 60 in-frame nucleotides (20,217 total loci). For mouse, we used the refFlat and knownGene transcript annotations from the UCSC mm9 genome assembly, and in the case of chicken, we used the refFlat and ensGene transcript annotations from the UCSC galGal3 genome assembly.

### Identifying Bidirectional Promoters

In agreement with previous studies, we defined BDPs as those regions of up to 1 kb long which are flanked by two head-to-head oriented transcripts. We first defined the set of BDPs flanked by two protein-coding transcripts (see above). To find PINT candidates, we searched for BDPs flanked by one protein-coding and one non-coding transcript (excluding any non-coding transcripts that overlap coding regions). Any such BDPs overlapping BDPs flanked by two protein-coding transcripts were excluded from the analysis.

### Identifying Lineage-specific Transcripts

For the purpose of identifying primate and human specific transcripts generated from BDPs, we identified BDPs flanked by one protein-coding and one non-coding transcript, for which only the protein-coding gene is conserved in mouse (i.e. the “anchor”). Non-coding transcripts were considered RefSeq and knownGene transcripts that lacked annotated ORFs. We additionally considered EST annotations, but only those from the intronEst set, which by the virtue of them being spliced, minimize the possibility of including short-lived RNA molecules. To identify the protein-coding genes conserved in mouse, we mapped locations of orthologous protein-coding genes in the mm9 UCSC mouse assembly using the UCSC liftOver tool, and considered genes conserved if at least one protein-coding transcript was transcribed on the same strand within a 500 bp window centered on the position corresponding to the mapped human TSS. Additionally, we required that the proteins corresponding to the human and mouse loci were identified as orthologs through a reciprocal best BLAST hit approach. We then scanned the upstream region of the mouse orthologs and discarded any cases where a transcript was found on the opposite strand with a TSS closer than 1 kb.

### Evaluation of Transcript Expression

To evaluate the correlation of expression between transcripts flanking BDPs, we used the Affymetrix exonic array data for 11 tissues (breast, cerebellum, heart, kidney, liver, muscle, pancreas, prostate, spleen, testes, and thyroid) available in the affyExonTissues table of the UCSC hg18 Table Browser. We used only probes mapping to the first exons (probes matching more than half of their size to TEs were excluded), which has the advantage of avoiding the influence of internal alternative promoters, where they exist [29]. We assigned each transcript an expression profile based on the ranking of probe median expression values in 11 tissues. The similarity of expression between two transcripts was evaluated with a parameter called relative expression difference (RED) that compares the ranks of the 11 tissues with the following formula:
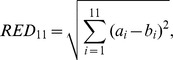
where *i* represents the tissue, and *a* and *b* represent the rank in tissue *i* of the upstream and downstream transcripts, respectively. The RED parameter was only computed for BDPs where both transcripts had at least one Affymetrix probe mapped to their first exon (694 total pairs). In the case that more than one probe was found matching the first exon, RED values for all possible pairs were computed and the lowest RED value was reported. Distributions of expected RED values were computed both with pairs of randomly selected transcripts, as well as with randomly selected pairs of adjacent transcripts. For the former, we selected 694 pairs of transcripts, where the first represented a randomly chosen protein-coding transcript, which we paired with a randomly chosen non-coding transcript from the same chromosome but from the opposite strand. The number of such pairs from each chromosome matched the chromosomal distribution of PINT-anchor pairs. The process was repeated 1,500 times, for a total of 1,041,000 pairs. In the case of randomly selected adjacent pairs, we first defined all loci containing non-coding transcripts. Transcripts that shared at least one splice junction (either acceptor or donor) were combined into the same locus. Then, for each of the 20,217 protein-coding loci (see above) we found the closest non-overlapping non-coding locus, and retained only those pairs of anti-sense loci for which at least one transcript per locus had Affymetrix probes matched to their first exons. Loci with intergenic distances shorter than 1 kb were excluded from the analysis. From these, we randomly selected 694 transcript pairs (with the same chromosomal distribution as the PINT-anchor pairs), repeating the process 1,500 times. The same procedures were followed for pairs of protein-coding loci.

### Analysis of Sequence Conservation

The conservation of transcript exonic sequences was evaluated with phastCons scores [50] determined from 17 way alignments of vertebrate species available through the Galaxy [51], [52] toolbox (http://galaxy.psu.edu). To determine the average phastCons score of a transcript we first determined average phastCons scores for all individual exons using the Aggregate datapoints (version 1.1.3) tool. The average transcript phastCons score was computed by averaging individual exon values weighted by exon sizes. Conservation of splice sites was computed in a similar way, with the coordinates of the “GT” (donor) and “AG” (acceptor) dinucleotides being used as input for the Aggregate datapoints tool. No transcript averages were computed for splice sites.

### Transposable Element Data

For human, we used the TE annotations contained in the pre-masked dataset for the hg18 UCSC assembly constructed with RepeatMasker version 3.2.7 and TE libraries from January 20, 2008 (http://www.repeatmasker.org/PreMaskedGenomes.html). This allows for the inspection of individual alignments between TE consensi and genomic sequences. In the case of mouse and chicken, we used the TE annotation associated with the UCSC mm9 and galGal3 genome assemblies.

### Evaluation of Splice Signal Strength

To evaluate the strength splicing consensus dinucleotides (“AG” and “GT” dinucleotides for acceptor and donor signals, respectively), we used the stand-alone version of SplicePort [33]. To compute a score, SplicePort uses 162-bp sequence centered on the dinucleotide, which we obtained by extracting from the corresponding genome 80 bps on each side of the dinucleotide.

### Estimating the Expected Fraction of TE-derived SSs

To calculate the expected versus observed fraction of TE-derived acceptor SSs for every set of transcripts (e.g. PINTs, anchors, cncRNAs), we first constructed a distribution of SplicePort scores for actual acceptor SSs used by transcripts in each set. We then scored all “AG” dinucleotides available at corresponding genomic loci and determined whether they overlap annotated TEs. To determine the expected fraction of TE-derived SSs, we randomly sampled from the genomic “AG” dinucleotides the same number of dinucleotides as in the actual set of SSs used by transcripts in each set, requiring the SplicePort score distribution of randomly sampled dinucleotides was similar to the score distribution of actual SSs (the distribution was imposed on bins of size 0.1). The procedure was repeated 10,000 times, each time counting the number of dinucleotides that overlap annotated TEs. The expected fraction of TE-derived SSs was the median of the distribution constructed with 10,000 values. We used the same distribution to determine whether the observed fraction of TE-derived SSs represents an extreme value (i.e. is located within the distribution tails and corresponding to an alpha level of 5%).

### Identifying Regions Subject to Adaptive Evolution

To identify regions subjected to forces of adaptive evolution, we first identified TE fragments in PINTs that have rates of human-specific substitution higher than 2%. For this purpose, we used 3-way human-chimp-macaque alignments available from the Galaxy web site (http://galaxy.psu.edu). To decide whether the observed rate of human-specific substitutions was significant, we constructed an empirical distribution of human-specific substitution rates using TE fragments homologous to each specific TE fragment of interest. To find specific TE homolog fragments, we first determined the specific region in the TE consensus sequence that corresponds to the TE fragment of interest, as provided with the RepeatMasker alignment files for the pre-annotated hg18 assembly. We then identified all TE fragments from intergenic regions that correspond to the exact coordinates in the consensus TE sequence. The human-specific substitution rate for each fragment was computed as an excess over the local rate of human-specific substitutions. The local rate was evaluated in a 4 kb region centered on the TE fragment (2 kb upstream, and 2 kb downstream), to account for biases introduced by potentially increased local recombination rates. We retained only the TE fragments with more than 1 kb of aligned sequence within the 4 kb flanking regions. With the values obtained from all intergenic homolog TE fragments, we built the empirical distribution for the expected human-specific substitution rate, which we then used to evaluate the significance the human-specific substitution rate observed for the PINT-embedded TE fragments. Fragments with less than 100 homolog copies were discarded.

### RT-PCR Survey of Human Tissues

To verify the splicing pattern of the third exon of the AK094354 transcript, we surveyed commercial RNA samples (Origene; 1 mg/ml) from 12 human tissues: brain, spleen, muscle, placenta, heart, liver, lung, stomach, kidney, intestine, testis, colon. cDNA was obtained from 1 µg of RNA by reverse transcription in a 20 µl reaction mix with the iScript™ cDNA synthesis kit (Bio-Rad Laboratories) as in the case of the minigene splicing assay (see Supplementary Materials). The cDNA was amplified in a 50 µl PCR reaction with primers designed to match the first(5′- TTGTTGGCAAACAGTTCTGGG-3′) and fourth(5′- CCAGACCATCACAAGGATATC-3′) exons of the AK094354 transcript. The PCR reaction mix consisted of 2 µl of cDNA template and 48 µl master mix with the following quantities for one reaction: 5 µl of 10× PCR buffer (15 mM MgCl_2_, Applied Biosystems), 0.5 µl (2.5 U) AmpliTaq® DNA polymerase (Applied Biosystems), 1 µl 10 mM dNTP, 1 µl of each of the two primers at 12.5 µM, 1 µl DMSO, 38.5 µl H_2_O. Conditions were set at 95°C denaturation for 5 min, followed by 32 cycles of 95°C for 30 s, 55°C for 45 s, 75°C for 1 min, with a final 7 min hold at 72°C. Products of the PCR reactions were visualized by gel electrophoresis on a 2% agarose gel.

## Supporting Information

Figure S1 **The chromosomal distribution of PINTs in the human genome (red) is not significantly different (** ***P*** **= 0.061, goodness-of-fit χ^2^ test) from what can be expected based on the distribution of potential anchor genes (gray).** In this case, potential anchor genes (a total of 11,015) were considered all protein-coding genes that are conserved in mouse and have at least 20 kb of their 5′ upstream region free of other protein coding genes.
(PDF)

Figure S2 **The RED parameter successfully detects expression correlation between protein-coding genes controlled by BDPs.** (**A**) The RED values associated with protein-coding genes controlled by BDPs (971 pairs; green) is significantly lower than RED values calculated for pairs of randomly selected protein-coding genes from opposite strands (light gray; *P* = 3.9×10^−8^, Wilcoxon rank sum test), and from RED values computed for random pairs of adjacent protein-coding transcripts (dark gray; *P* = 0.0043). For each of the two random distributions, 1,000,130 pairs of transcripts were used (1,030 sets of 971). (**B**) The significance of differences is highlighted by comparing the median RED value associated with the BDP-controlled protein-coding genes (11.92; green arrow) with the distribution of median RED values calculated for the 1,030 sets of random pairs: median 12.49 for pairs of randomly selected transcripts (light gray; *P*<9.7×10^−4^), and median 12.17 for random pairs of adjacent transcripts (dark gray, *P* = 0.023).
(PDF)

Figure S3 **The acceptor SS occurrence in TE-derived sequences was also evaluated in a set of BDP-flanking 400 non-coding transcripts (cncRNAs) with transcriptional activity at the mouse orthologous locus.** SplicePort score distributions of all splice signals found at the 400 genomic loci (black) and the actual acceptor SSs (green) are shown in the top panel, while the expected fraction of signals residing in TE-derived sequences in shown in the bottom panel (solid black). The vertical green line corresponds to the median score associated with the 771 acceptor SSs (0.412). The horizontal dotted green line corresponds to the fraction of actual SS residing in TE-derived sequences (29.8%), which is significantly lower (*P*<10^−4^) than the expected 35.6% value.
(PDF)

Figure S4 **TE frequencies around the 3′ end of anchor genes reveals no region with preferential TE accumulation.** Anchor regions are shown in blue, regions downstream of anchor 3′ end are shown in black, and genomic average values (computed over 10,000 randomly selected 400-kb regions) are shown in grey.
(PDF)

Figure S5 **The distribution of TE frequency around the TSS of human protein-coding genes.** Blue corresponds to genomic loci occupied by and downstream of protein-coding genes (a total of 20,217 loci), while black corresponds to levels observed upstream of protein-coding genes. The gray line indicates background TE levels as computed in 20,000 randomly selected genomic segments around protein-coding genes. It is obvious that the region just upstream of the promoter region exhibit preferential TE accumulation.
(PDF)

Figure S6 **The distribution of TE frequency around the TSS of mouse protein-coding genes (a total of 20,837 loci).** Similarly to the TE profile in human, a region of preferential TE accumulation emerges just upstream of the promoter regions.
(PDF)

Figure S7 **The distribution of TE frequency around the TSS of chicken protein-coding genes (a total of 14,273 loci).** Similarly to the TE profile in human and mouse, a region of preferential TE accumulation emerges just upstream of the promoter regions.
(PDF)

Figure S8 **The distribution of primate-specific Alu elements around the TSS of human protein-coding genes.** The preferential accumulation upstream of the promoter region is more accentuated then in the case of the profile built with all TEs.
(PDF)

Figure S9 **The distribution of rodent-specific B1 elements around the TSS of mouse protein-coding genes.** The preferential accumulation upstream of the promoter region is more accentuated then in the case of the profile built with all TEs.
(PDF)

Figure S10 **The distribution of chicken-specific CR1 elements around the TSS of chicken protein-coding genes.** The profile is very similar to the profile built with all TEs, indicating that the effect is due to the most active chicken TEs, the CR1 LINE.
(PDF)

Figure S11 **Length distribution of the longest ORFs found in PINT sequences. In 35 cases (3.2%), no valid ORF was found.**
(PDF)

Figure S12 **Evidence of protein-coding potential for the ORFs found in PINT sequences as evaluated by the Coding Potential Calculator (CPC;** http://cpc.cbi.pku.edu.cn/ **).** (**A**) Distribution of the composite CPC scores. The CPC score is assigned using evidence from multiple sources including matches to other known or annotated ORFs, frame and coverage of matches, log-odds scores, and scores above 0 indicate protein-coding potential (14.5% of cases). (**B**) The distribution of log-odds scores suggests that only a smaller fraction (1.4%) of ORFs have the potential to encode functional proteins (values above 60 are considered to correspond to protein-coding sequences).
(PDF)

Figure S13 **Profiles of weak-to-strong (W->S) substitution bias around 32 TE fragments found to evolve at accelerated rates in the human lineage.** Two graphs are provided for each TE fragment (coordinates, size, name and class/family of each accelerated TE fragment are provided above set of graphs): *i*) proportion of W->S mutations computed for windows of 20 mutations (centered on each mutation). Each human-specific mutation in the shown interval is represented by a dot on the graph. The red line denotes the 50% mark; position 0 indicates the center of the accelerated TE fragment; *ii*) values of the *G* function [53] computed for W->S mutations. Hotspots of W->S mutations are highlighted by monotonically increasing *G*. The shaded central region of the graph corresponds to the location of the TE fragment. Examples of regions strongly affected by GC-biased gene conversion are provided in Fig. S14.
(PDF)

Figure S14 **Profiles of weak-to-strong (W->S) substitution bias around two typical regions shown to be affected by biased gene conversion: the** ***ADCYAP1*** **gene** [**38**] **and the HAR1 element** [**54**] **. Graphs were constructed the same way as those in Fig. S13.**
(PDF)

Figure S15 **Sizing and quantification results for PCR products detected in the minigene splicing assay.** The graphs correspond to gel lanes shown in Fig. 6A: human (A), chimp (B), macaque (C), chimp exon and human flanks (D), human exon and chimp flanks (E). The x axis in each graph indicates the fragment length, and the y axis the fluorescent intensity measured in Relative Fluorescent Units (RFU). Peaks in graphs correspond to the bands observed on the gel, with the first number below indicating the band size (bps) and the second indicating its fluorescent intensity (RFU).
(PDF)

Figure S16 **Lineage-specific novel transcripts are significantly more likely to emerge in close proximity of active promoters than in other genomic regions.** Distribution in blue was constructed through random sampling of 19,472 regions located away from promoters and counting in how many of them potential novel transcripts can be found (10,000 total replicates). The arrow in blue indicates the median of this distribution (227), while the arrow in blue corresponds to the number of PINTs. The comparison is conservative, because the expected value of 227 is an overestimate due to relaxed conditions imposed to finding potential transcripts.
(PDF)

Figure S17 **Testes and cerebellum are the tissues with the highest fraction of most highly expressed PINTs.** The distributions of the highest expressing tissue for PINTs and their anchors are shown in red and blue, respectively. Distributions shown in lighter shades correspond to random sets (1,000 replicates) of non-coding and coding transcripts, respectively (error bars correspond to standard deviation values). Expression is evaluated using only Affymetrix probes matching the first exons of transcripts.
(PDF)

Table S1 **Validation of BDP activity through RT-PCR of flanking genes.** Within the GENCODE framework [25], PCR primers were designed to amplify transcripts from both the upstream and downstream gene loci flanking a BDP (brain, heart, kidney, liver, lung, muscle, spleen, testis). The activity of a BDP in a given tissue was validated if expected PCR product sizes were detected from both gene loci in that respective tissue. The activity of 34 BDPs was validated in at least one tissue, primer design failed in one case, and the activity of five BDPs could not be validated in any of the eight tissues tested.
(PDF)

Table S2 **TE fragments in PINT exons found to have accelerated rates of evolution in the human lineage.** Table columns correspond to the following: **A** – coordinates of the TE fragment. [ ] denote a fragment that encompasses a shorter fragment (row 2), but it is part of a transcript on the reverse complement strand (included here to highlight the significance of the three human-specific mutations even in the context of a larger fragment); **B** – TE fragment length (bps); **C** – TE name; **D** – TE class/family; **E** – TE consensus coordinates corresponding to the TE fragment (parentheses indicate a match to the reverse complement strand); **F** – length of human-chimp-macaque ungapped alignment (bps); **G** – number of human-specific substitutions; **H** – rate of human-specific substitutions (%); **I** – excess of human-specific substitutions over flanking regions, computed by subtracting the rate of human-specific substitutions observed in the 4 kb flanking regions (2 kb upstream of the TE fragment, and 2 kb downstream) from the rate observed in the TE fragment (%); **J** – *P*-value associated with the excess rate (column I) estimated from a distribution of excess rates computed for specific intergenic TE homolog fragments (column L). * denotes fragments located in significant hotspots of weak-to-strong (W->S) mutations; **K** – *P*-value adjusted for multiple testing (FDR), computed in the R package with the “p.adjust(method = “BH”)” command with the P-values computed for all 51 PINT TE fragments with human-specific rates of evolution greater than 2%; **L** – number of specific intergenic TE homolog fragments used for significance estimate (column J).
(PDF)

Table S3 **Sequences cloned into pUC57 vectors for transfections into K562 cells.** In addition to these three, two more sequences were created by swapping the human exon (70 bps between chr10∶127388057–127388126) for the chimp ortholog (chr10∶126680637–126680706).
(PDF)

Table S4 **SplicePort scores for the SSs of the AluSx-derived exon (hg18 coordinates chr22∶23178179–23178296) in the noncoding minor C22orf45 isoform.** The lack of consensus dinucleotides in macaque indicates that the splice sites were acquired in the hominoid lineage, but their splicing efficiency was diminished by three human-specific mutations.
(PDF)

Methods S1 (DOCX)
