# Supplementary figures and images for: Bidirectional Promoters as Important Drivers for the Emergence of Species-Specific Transcripts
Source: PLoS One. 2013 Feb 27;8(2):e57323. doi: 10.1371/journal.pone.0057323 (PMC3583895; doi:10.1371/journal.pone.0057323)

Figure S1

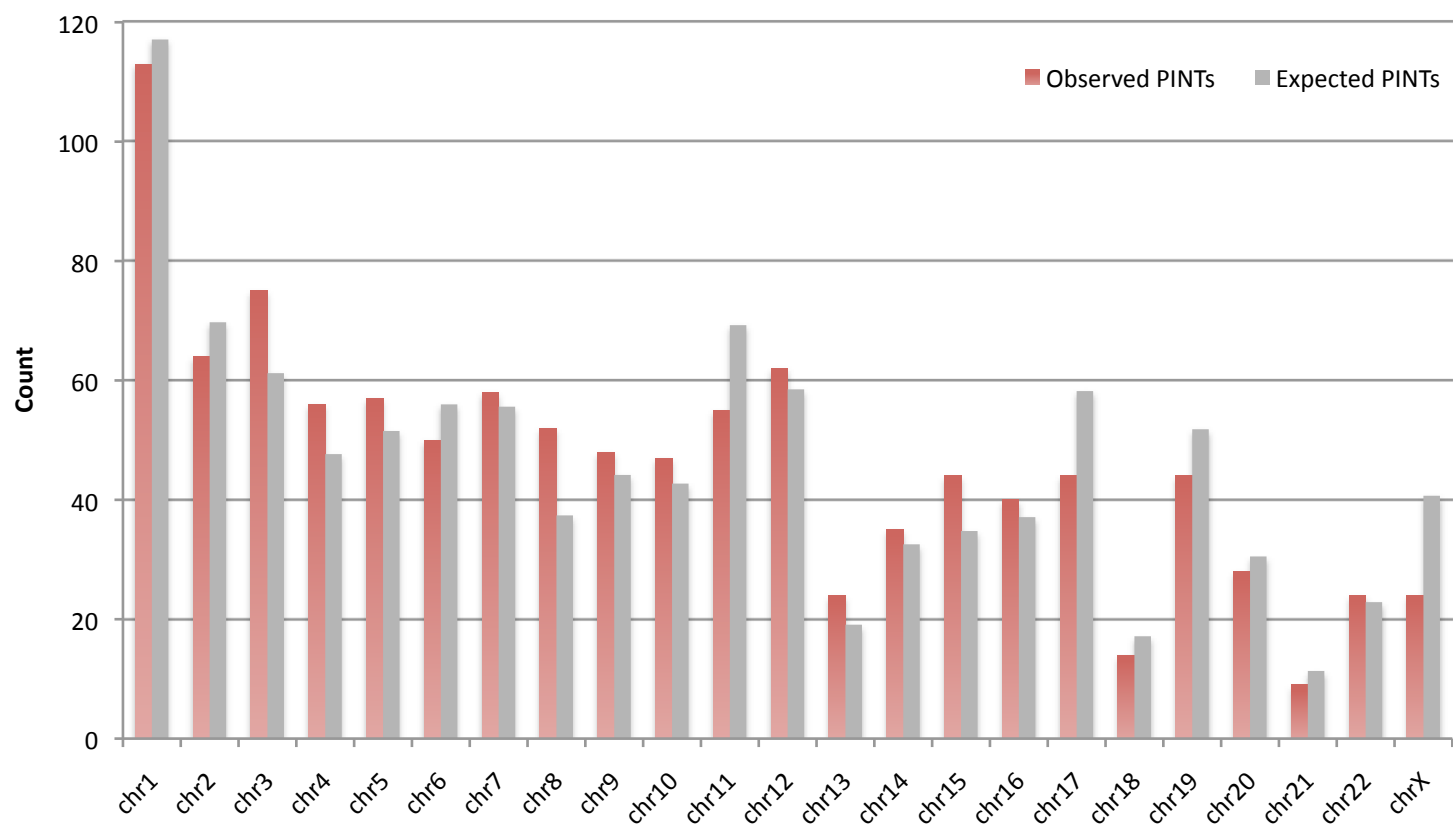

Supplement: Figure S1 — The chromosomal distribution of PINTs in the human genome (red) is not significantly different ( P = 0.061, goodness-of-fit χ2 test) from what can be expected based on the distribution of potential anchor genes (gray). In this case, potential anchor genes (a total of 11,015) were considered all protein-coding genes that are conserved in mouse and have at least 20 kb of their 5′ upstream region free of other protein coding genes. (PDF) [file pone.0057323.s001.pdf]

Figure S2

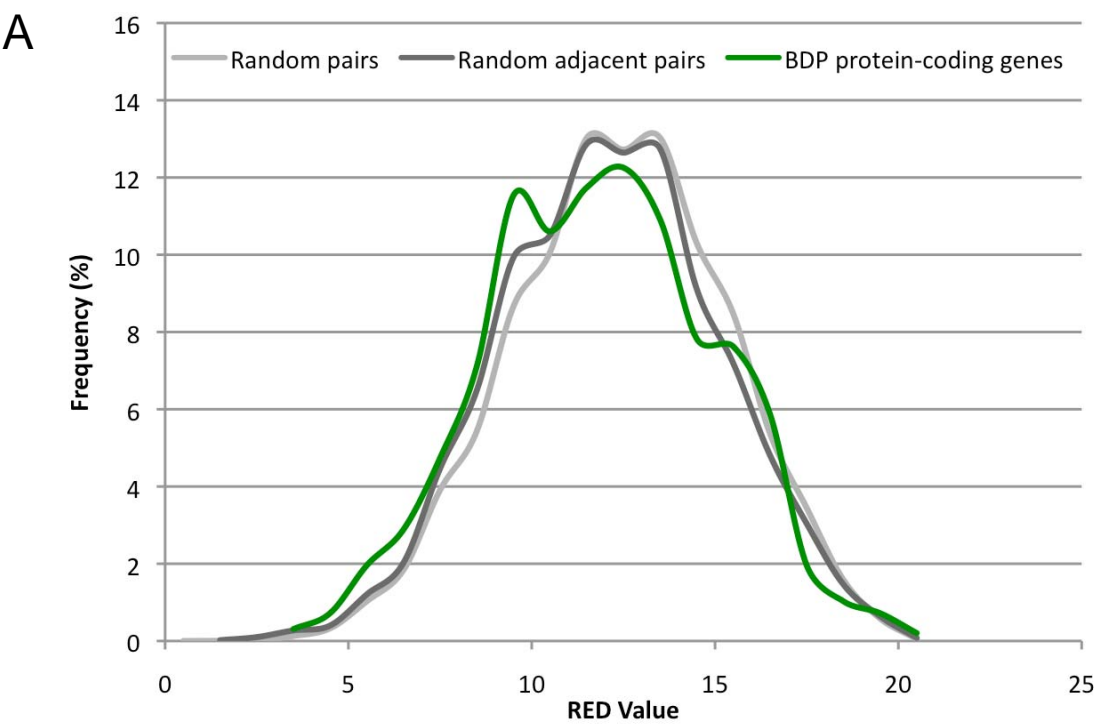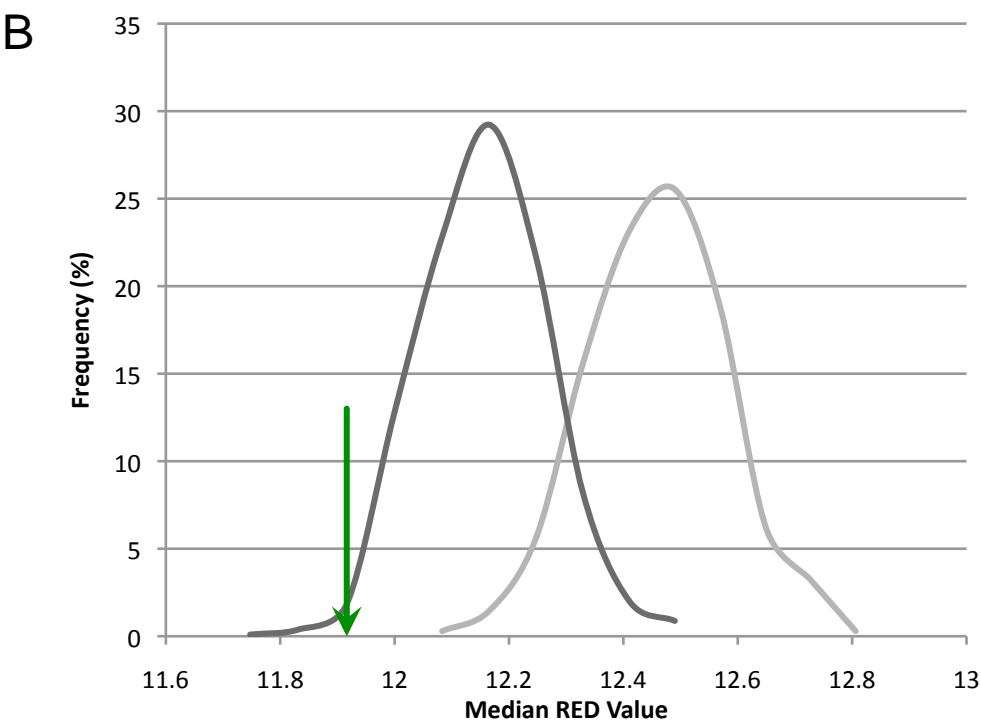

Supplement: Figure S2 — The RED parameter successfully detects expression correlation between protein-coding genes controlled by BDPs. (A) The RED values associated with protein-coding genes controlled by BDPs (971 pairs; green) is significantly lower than RED values calculated for pairs of randomly selected protein-coding genes from opposite strands (light gray; P = 3.9×10^−8^, Wilcoxon rank sum test), and from RED values computed for random pairs of adjacent protein-coding transcripts (dark gray; P = 0.0043). For each of the two random distributions, 1,000,130 pairs of transcripts were used (1,030 sets of 971). (B) The significance of differences is highlighted by comparing the median RED value associated with the BDP-controlled protein-coding genes (11.92; green arrow) with the distribution of median RED values calculated for the 1,030 sets of random pairs: median 12.49 for pairs of randomly selected transcripts (light gray; P<9.7×10^−4^), and median 12.17 for random pairs of adjacent transcripts (dark gray, P = 0.023). (PDF) [file pone.0057323.s002.pdf]

Figure S3

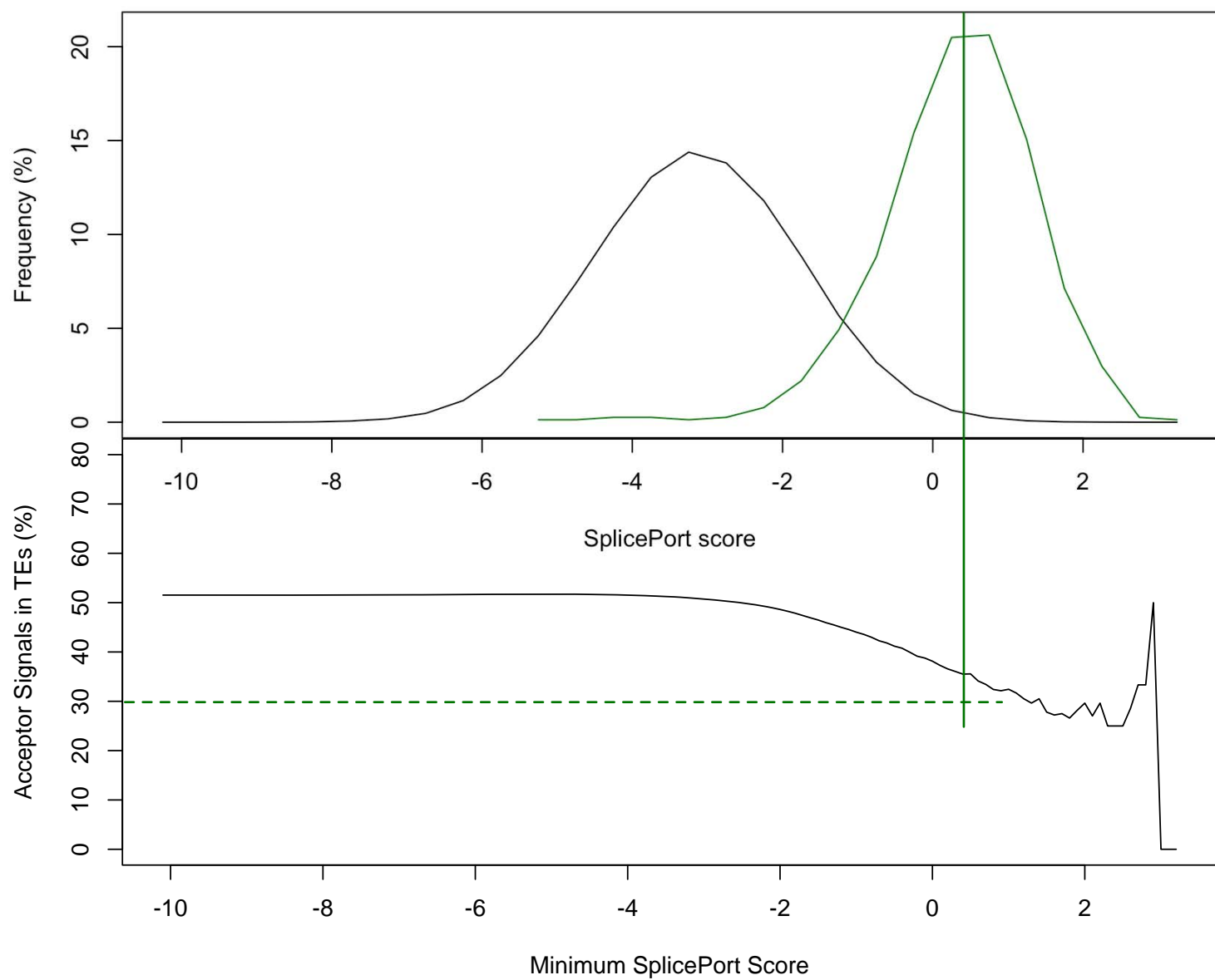

Supplement: Figure S3 — The acceptor SS occurrence in TE-derived sequences was also evaluated in a set of BDP-flanking 400 non-coding transcripts (cncRNAs) with transcriptional activity at the mouse orthologous locus. SplicePort score distributions of all splice signals found at the 400 genomic loci (black) and the actual acceptor SSs (green) are shown in the top panel, while the expected fraction of signals residing in TE-derived sequences in shown in the bottom panel (solid black). The vertical green line corresponds to the median score associated with the 771 acceptor SSs (0.412). The horizontal dotted green line corresponds to the fraction of actual SS residing in TE-derived sequences (29.8%), which is significantly lower (P<10−4) than the expected 35.6% value. (PDF) [file pone.0057323.s003.pdf]

Figure S4

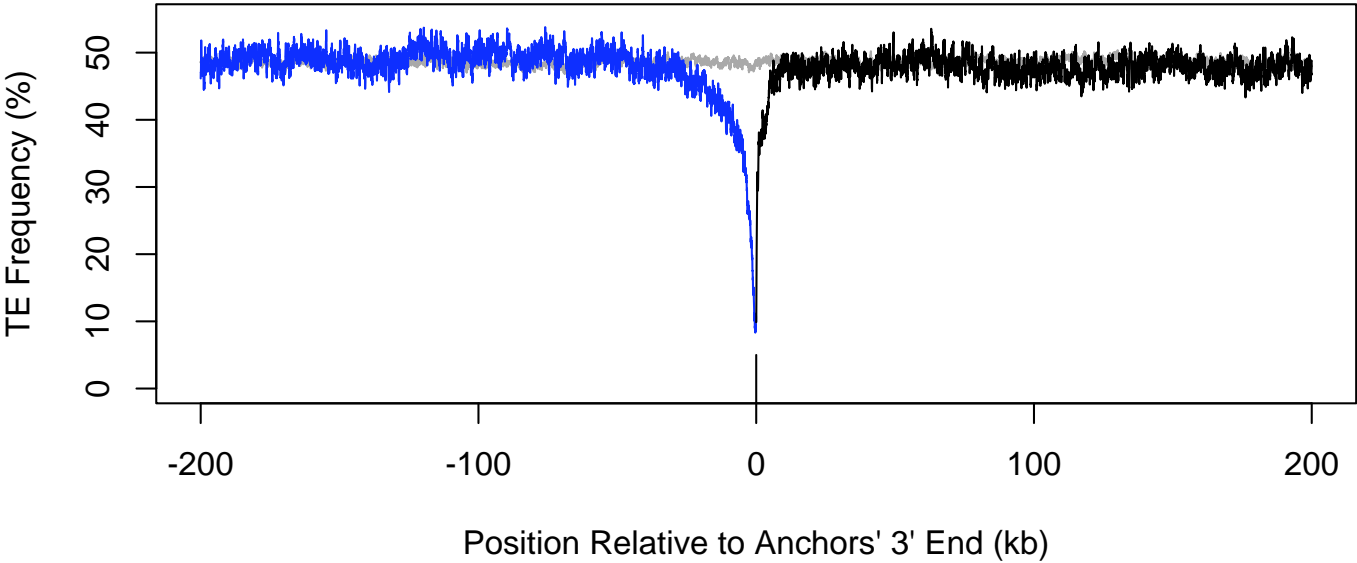

Supplement: Figure S4 — TE frequencies around the 3′ end of anchor genes reveals no region with preferential TE accumulation. Anchor regions are shown in blue, regions downstream of anchor 3′ end are shown in black, and genomic average values (computed over 10,000 randomly selected 400-kb regions) are shown in grey. (PDF) [file pone.0057323.s004.pdf]

Figure S5

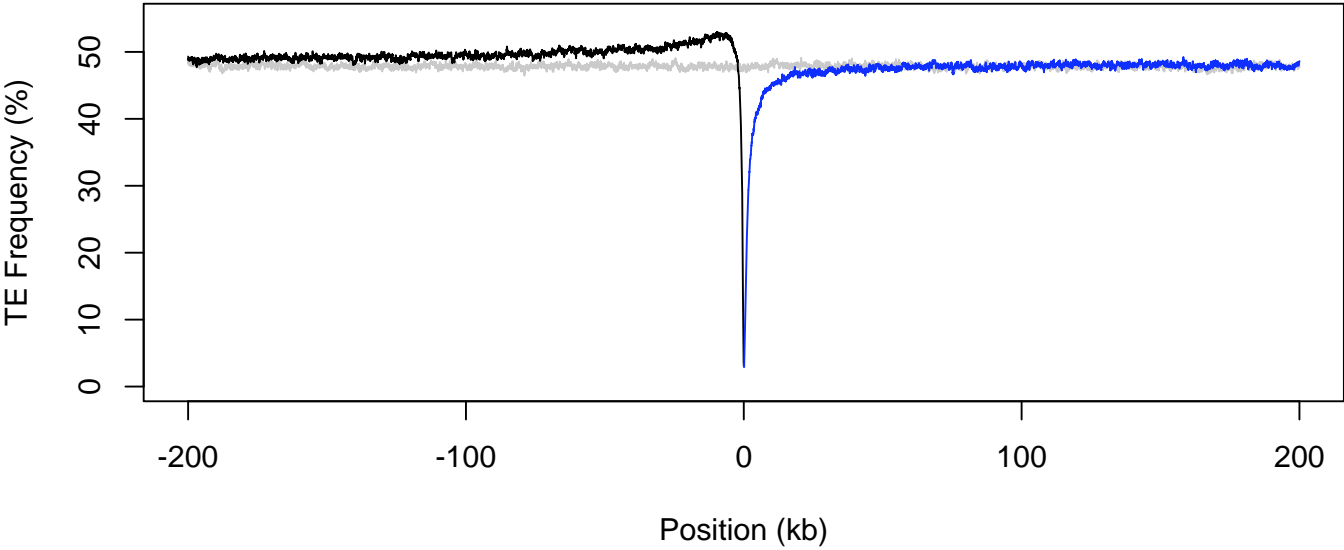

Supplement: Figure S5 — The distribution of TE frequency around the TSS of human protein-coding genes. Blue corresponds to genomic loci occupied by and downstream of protein-coding genes (a total of 20,217 loci), while black corresponds to levels observed upstream of protein-coding genes. The gray line indicates background TE levels as computed in 20,000 randomly selected genomic segments around protein-coding genes. It is obvious that the region just upstream of the promoter region exhibit preferential TE accumulation. (PDF) [file pone.0057323.s005.pdf]

Figure S6

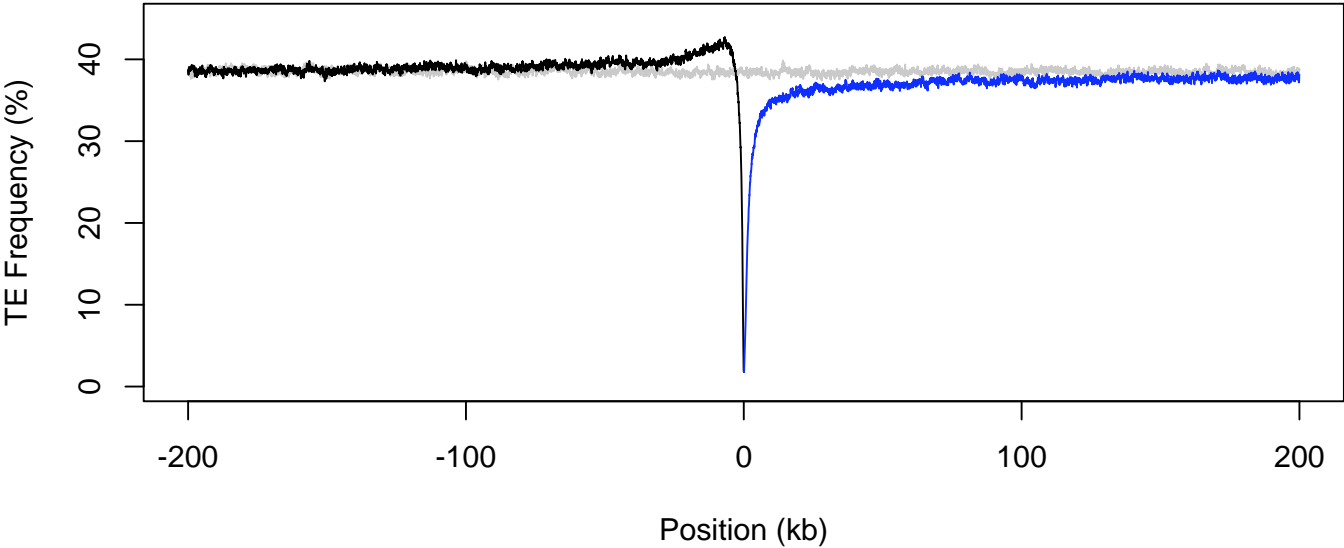

Supplement: Figure S6 — The distribution of TE frequency around the TSS of mouse protein-coding genes (a total of 20,837 loci). Similarly to the TE profile in human, a region of preferential TE accumulation emerges just upstream of the promoter regions. (PDF) [file pone.0057323.s006.pdf]

Figure S7

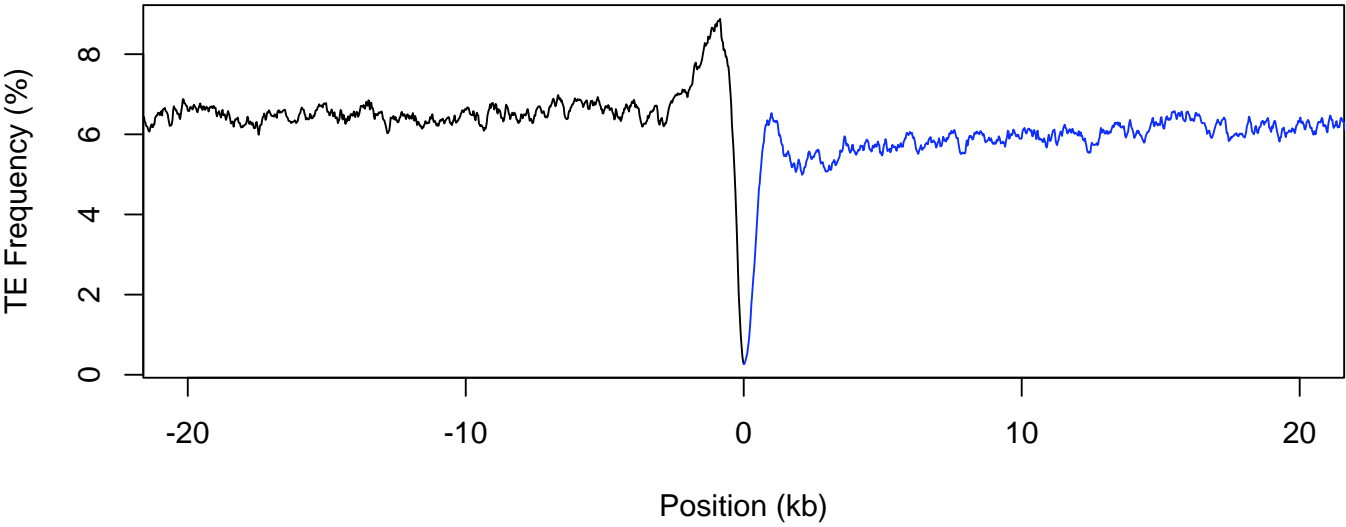

Supplement: Figure S7 — The distribution of TE frequency around the TSS of chicken protein-coding genes (a total of 14,273 loci). Similarly to the TE profile in human and mouse, a region of preferential TE accumulation emerges just upstream of the promoter regions. (PDF) [file pone.0057323.s007.pdf]

Figure S8

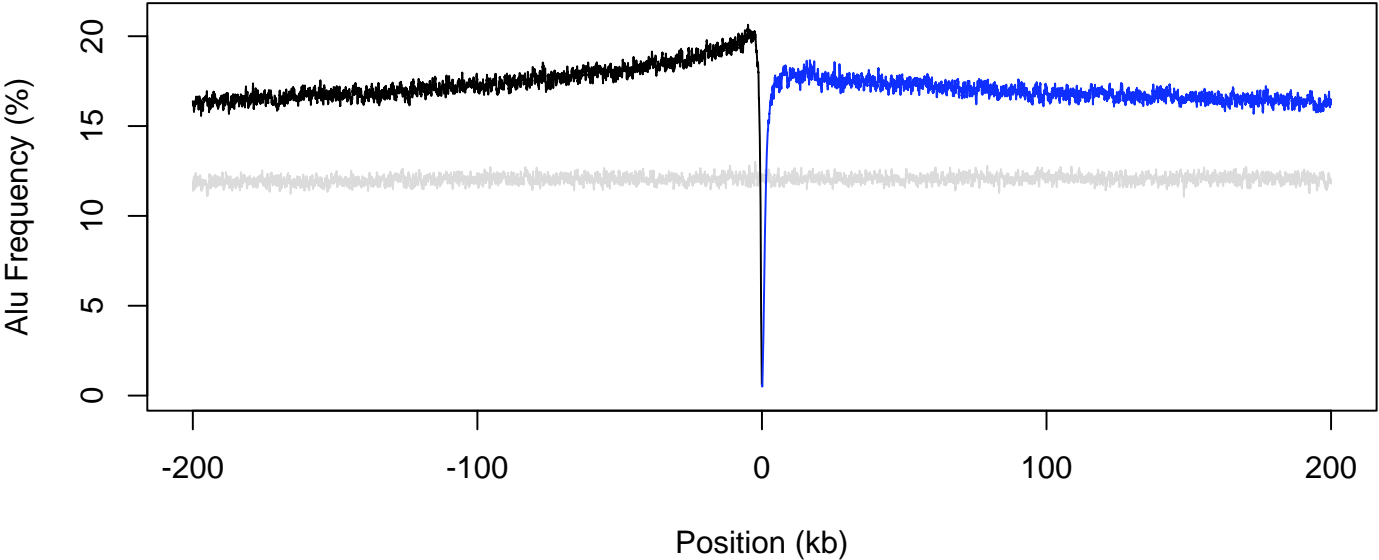

Supplement: Figure S8 — The distribution of primate-specific Alu elements around the TSS of human protein-coding genes. The preferential accumulation upstream of the promoter region is more accentuated then in the case of the profile built with all TEs. (PDF) [file pone.0057323.s008.pdf]

Figure S9

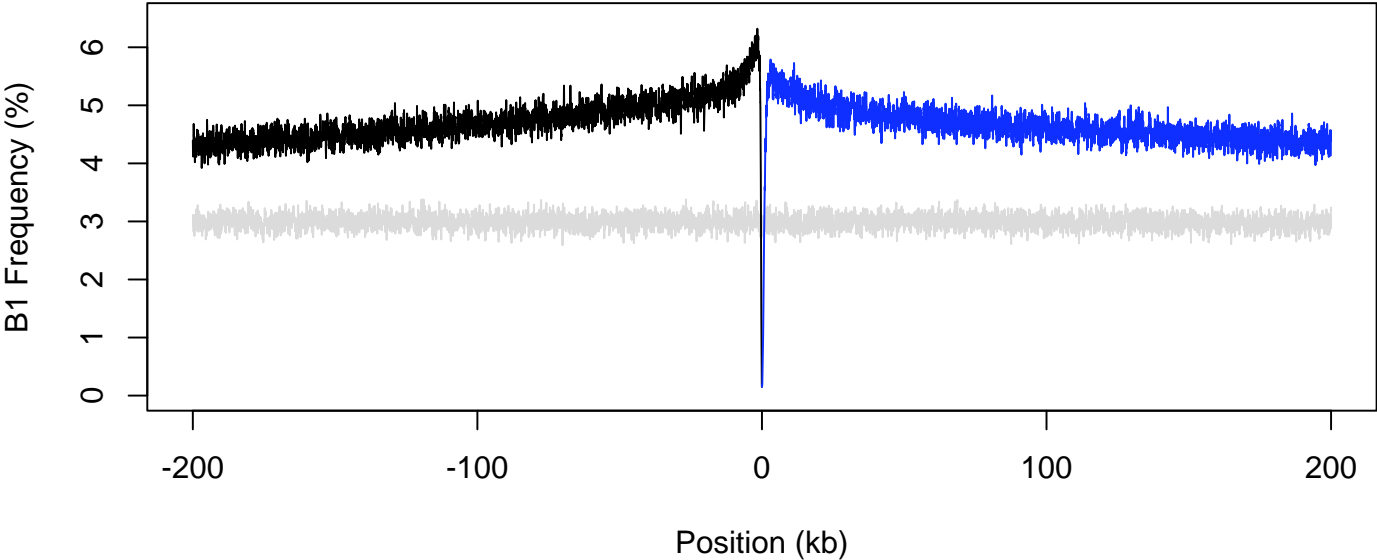

Supplement: Figure S9 — The distribution of rodent-specific B1 elements around the TSS of mouse protein-coding genes. The preferential accumulation upstream of the promoter region is more accentuated then in the case of the profile built with all TEs. (PDF) [file pone.0057323.s009.pdf]

Figure S10

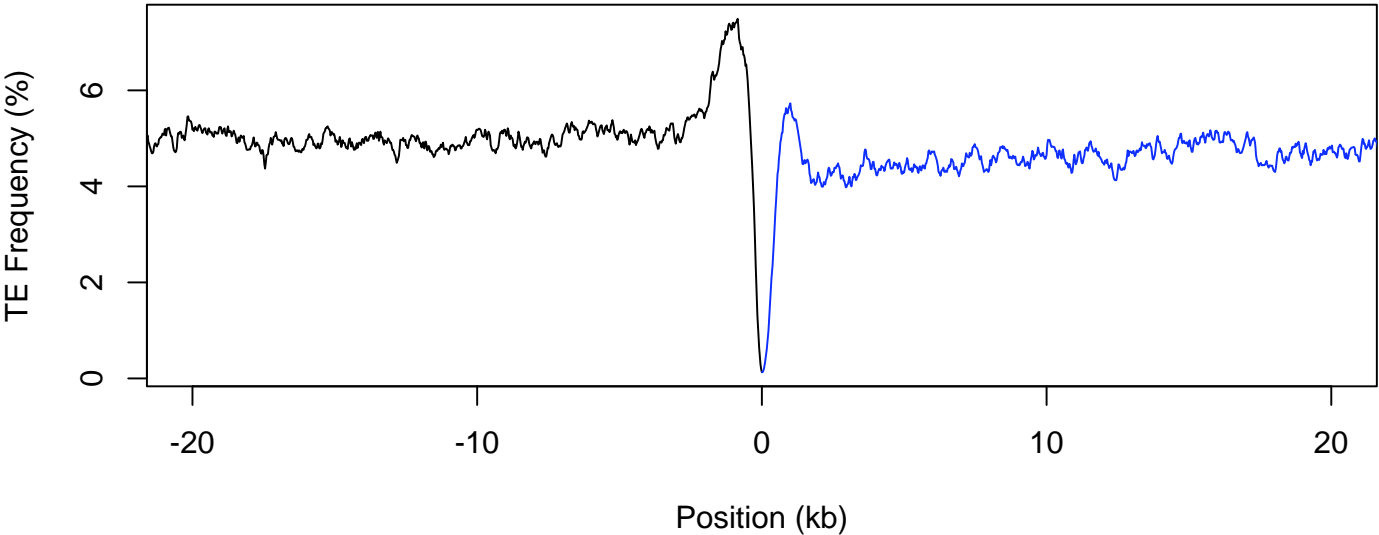

Supplement: Figure S10 — The distribution of chicken-specific CR1 elements around the TSS of chicken protein-coding genes. The profile is very similar to the profile built with all TEs, indicating that the effect is due to the most active chicken TEs, the CR1 LINE. (PDF) [file pone.0057323.s010.pdf]

Figure S11

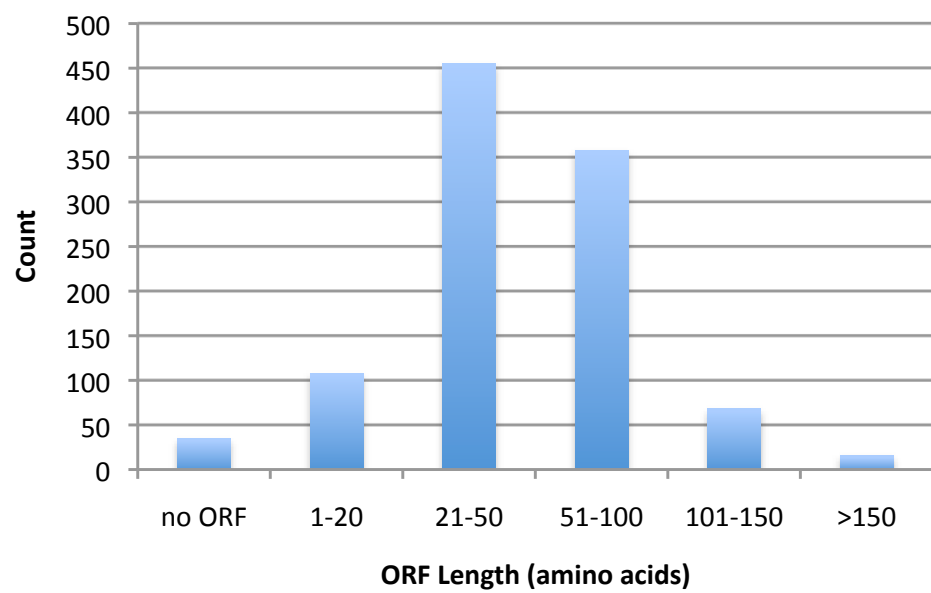

Supplement: Figure S11 — Length distribution of the longest ORFs found in PINT sequences. In 35 cases (3.2%), no valid ORF was found. (PDF) [file pone.0057323.s011.pdf]

Figure S12

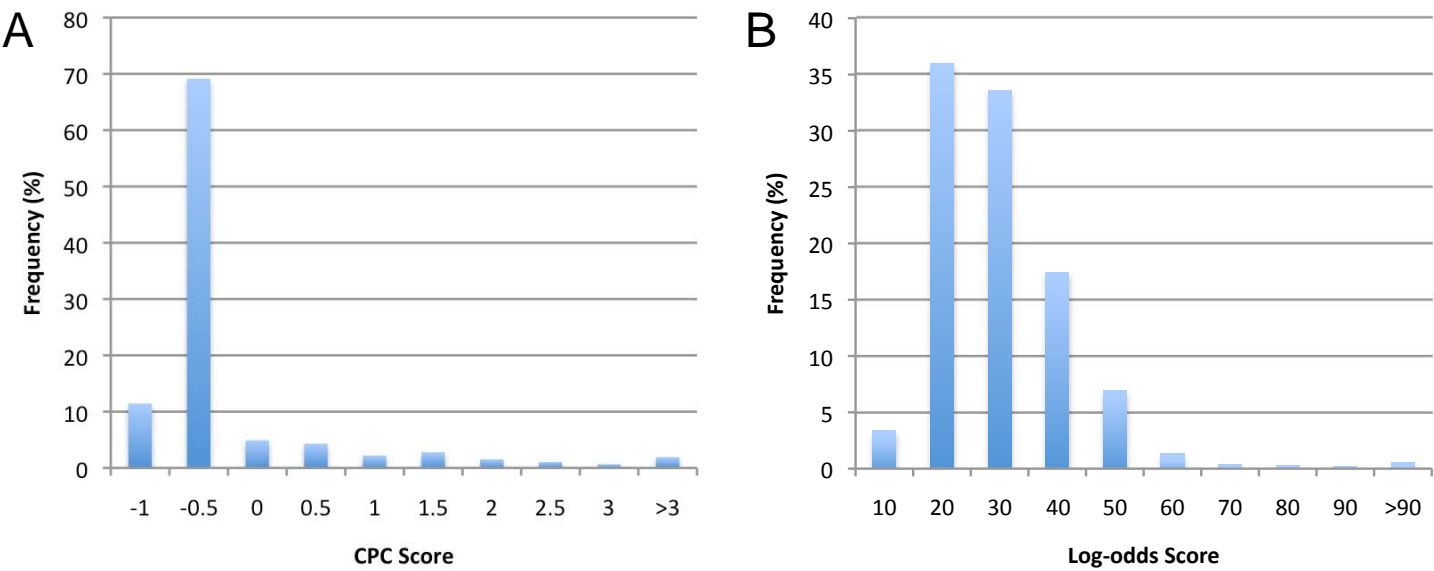

Supplement: Figure S12 — Evidence of protein-coding potential for the ORFs found in PINT sequences as evaluated by the Coding Potential Calculator (CPC; http://cpc.cbi.pku.edu.cn/ ). (A) Distribution of the composite CPC scores. The CPC score is assigned using evidence from multiple sources including matches to other known or annotated ORFs, frame and coverage of matches, log-odds scores, and scores above 0 indicate protein-coding potential (14.5% of cases). (B) The distribution of log-odds scores suggests that only a smaller fraction (1.4%) of ORFs have the potential to encode functional proteins (values above 60 are considered to correspond to protein-coding sequences). (PDF) [file pone.0057323.s012.pdf]

Figure S14

chr18:895388-899636 || 4249 || *ADCYAP1*

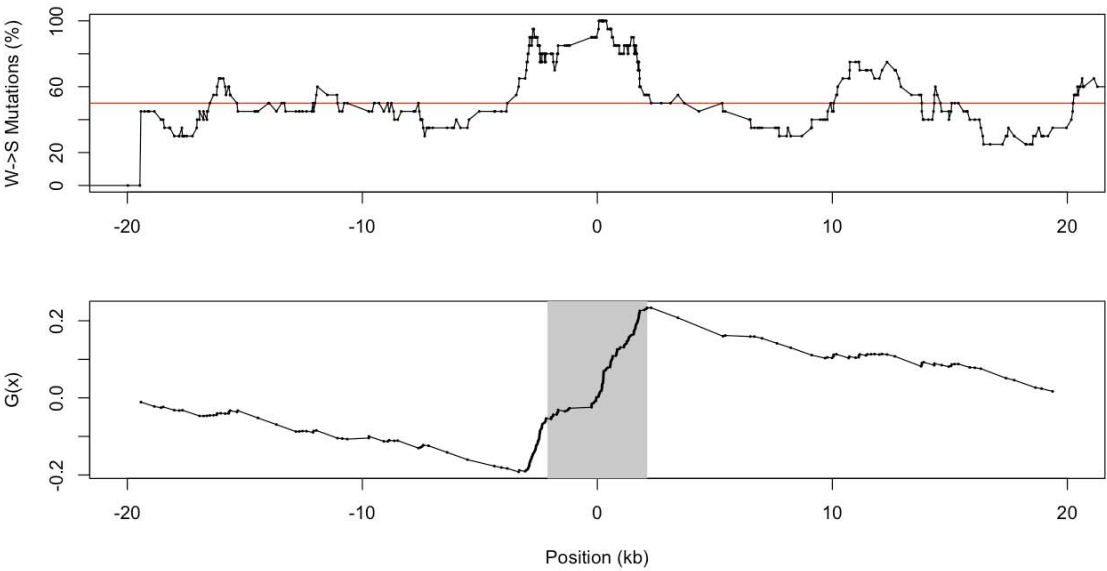

chr20:61203966-61204071 || 106 || *HAR1*

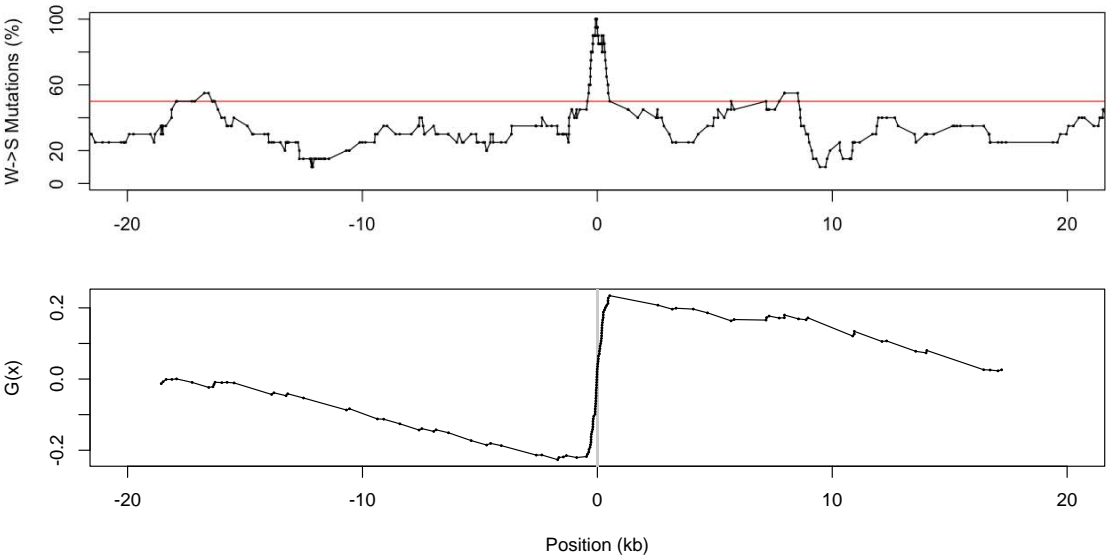

Supplement: Figure S14 — Profiles of weak-to-strong (W->S) substitution bias around two typical regions shown to be affected by biased gene conversion: the ADCYAP1 gene [38] and the HAR1 element [54] . Graphs were constructed the same way as those in Fig. S13. (PDF) [file pone.0057323.s014.pdf]

Figure S15

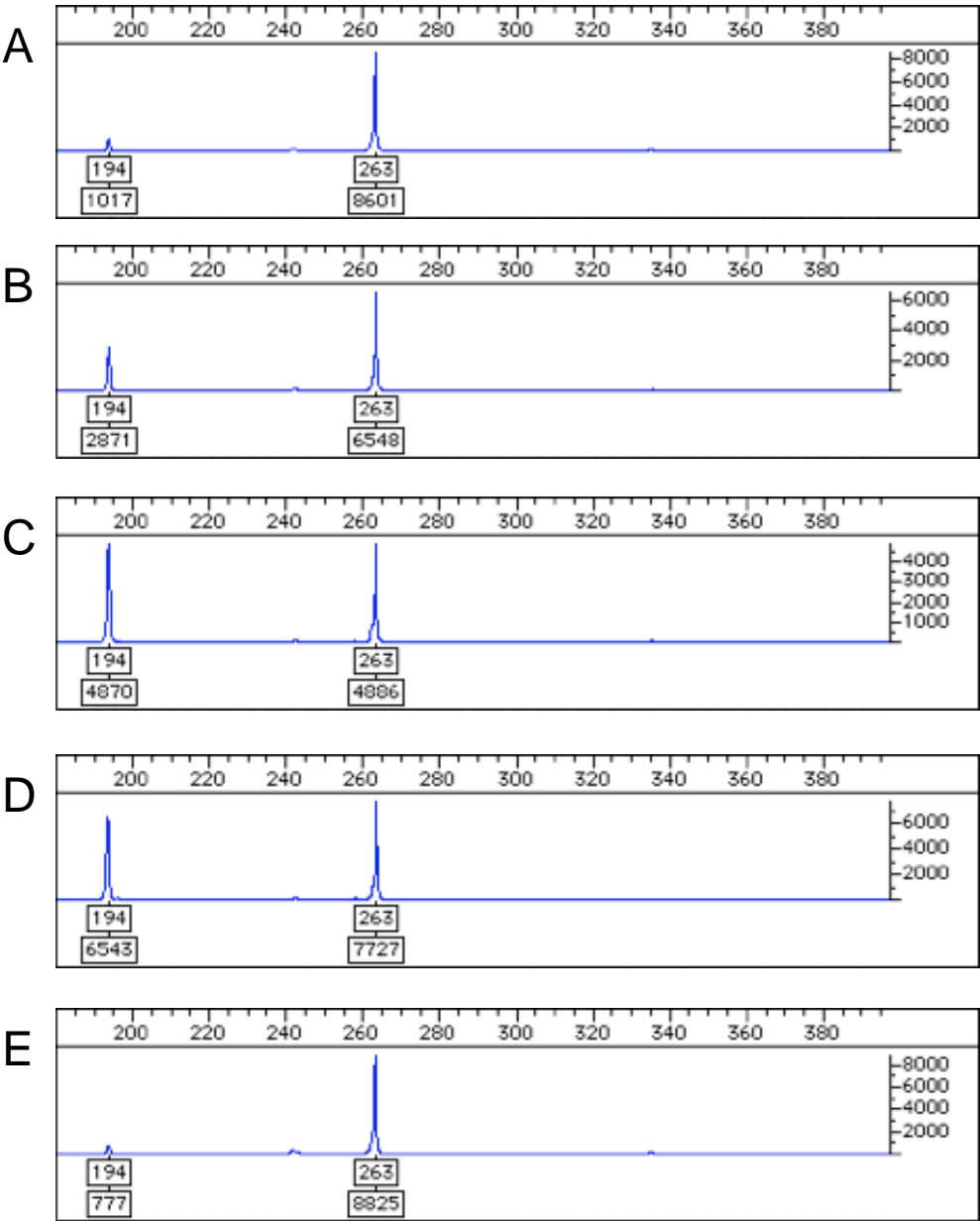

Supplement: Figure S15 — Sizing and quantification results for PCR products detected in the minigene splicing assay. The graphs correspond to gel lanes shown in Fig. 6A: human (A), chimp (B), macaque (C), chimp exon and human flanks (D), human exon and chimp flanks (E). The x axis in each graph indicates the fragment length, and the y axis the fluorescent intensity measured in Relative Fluorescent Units (RFU). Peaks in graphs correspond to the bands observed on the gel, with the first number below indicating the band size (bps) and the second indicating its fluorescent intensity (RFU). (PDF) [file pone.0057323.s015.pdf]

Figure S16

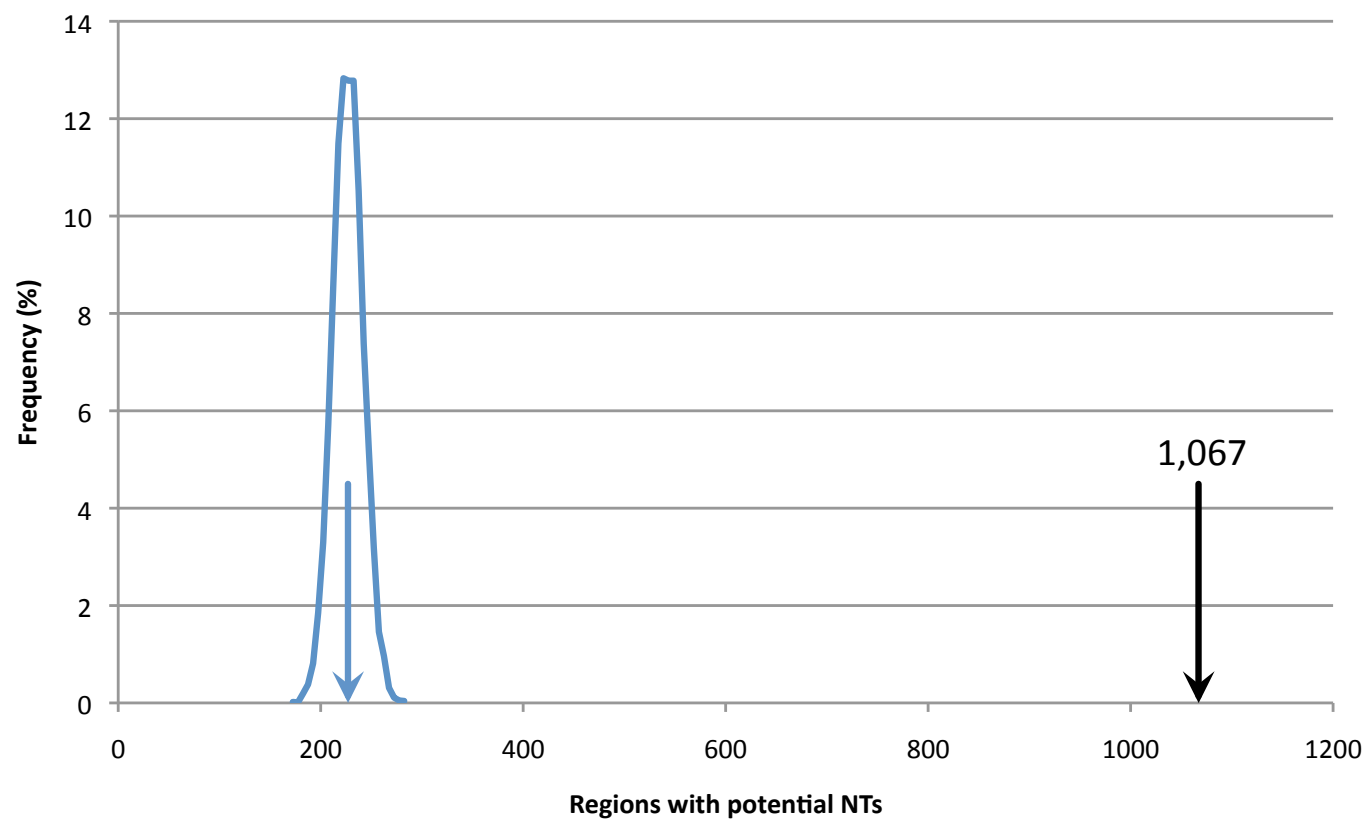

Supplement: Figure S16 — Lineage-specific novel transcripts are significantly more likely to emerge in close proximity of active promoters than in other genomic regions. Distribution in blue was constructed through random sampling of 19,472 regions located away from promoters and counting in how many of them potential novel transcripts can be found (10,000 total replicates). The arrow in blue indicates the median of this distribution (227), while the arrow in blue corresponds to the number of PINTs. The comparison is conservative, because the expected value of 227 is an overestimate due to relaxed conditions imposed to finding potential transcripts. (PDF) [file pone.0057323.s016.pdf]

Figure S17

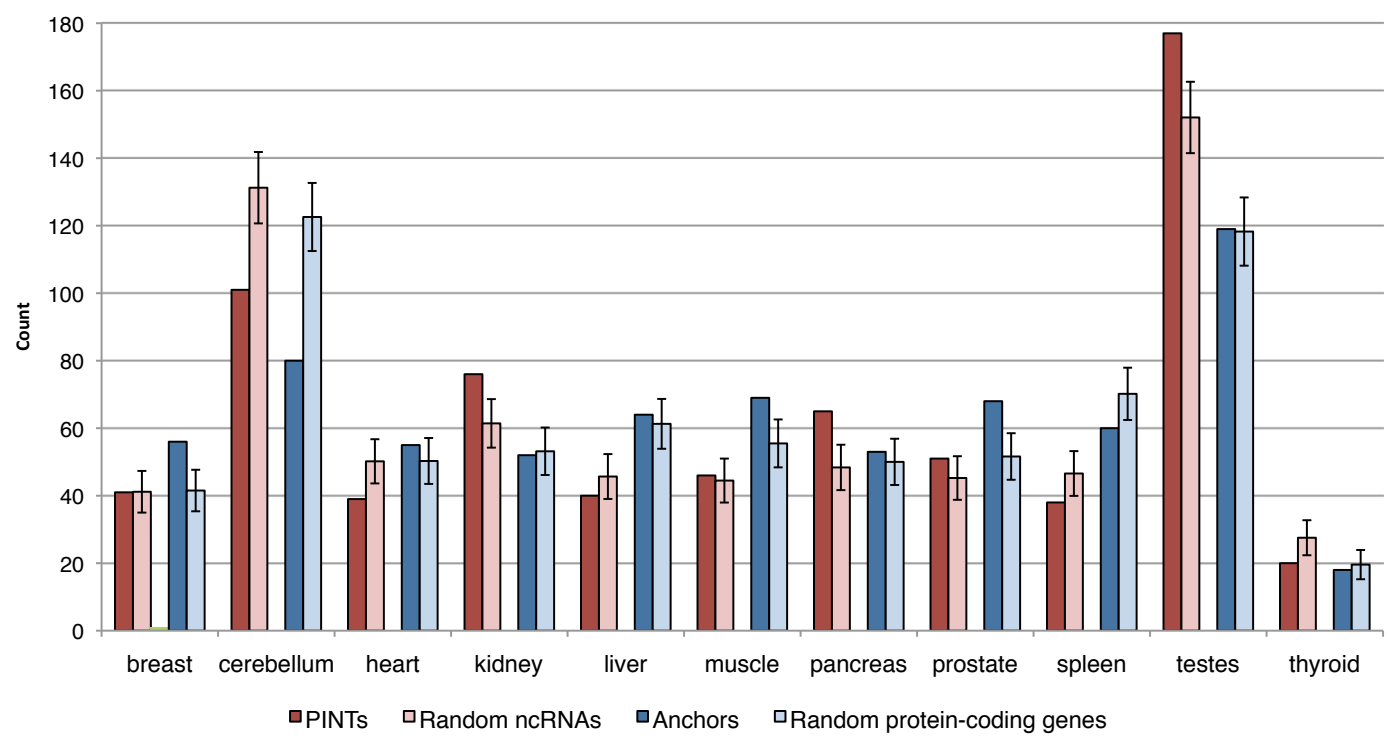

Supplement: Figure S17 — Testes and cerebellum are the tissues with the highest fraction of most highly expressed PINTs. The distributions of the highest expressing tissue for PINTs and their anchors are shown in red and blue, respectively. Distributions shown in lighter shades correspond to random sets (1,000 replicates) of non-coding and coding transcripts, respectively (error bars correspond to standard deviation values). Expression is evaluated using only Affymetrix probes matching the first exons of transcripts. (PDF) [file pone.0057323.s017.pdf]
