# Supplementary material for: Bidirectional Promoters as Important Drivers for the Emergence of Species-Specific Transcripts
Source: PLoS One. 2013 Feb 27;8(2):e57323. doi: 10.1371/journal.pone.0057323 (PMC3583895; doi:10.1371/journal.pone.0057323)

Figure S13

chr6:32743252-32743430 || 179 || Zaphod || DNA/hAT-Tip100

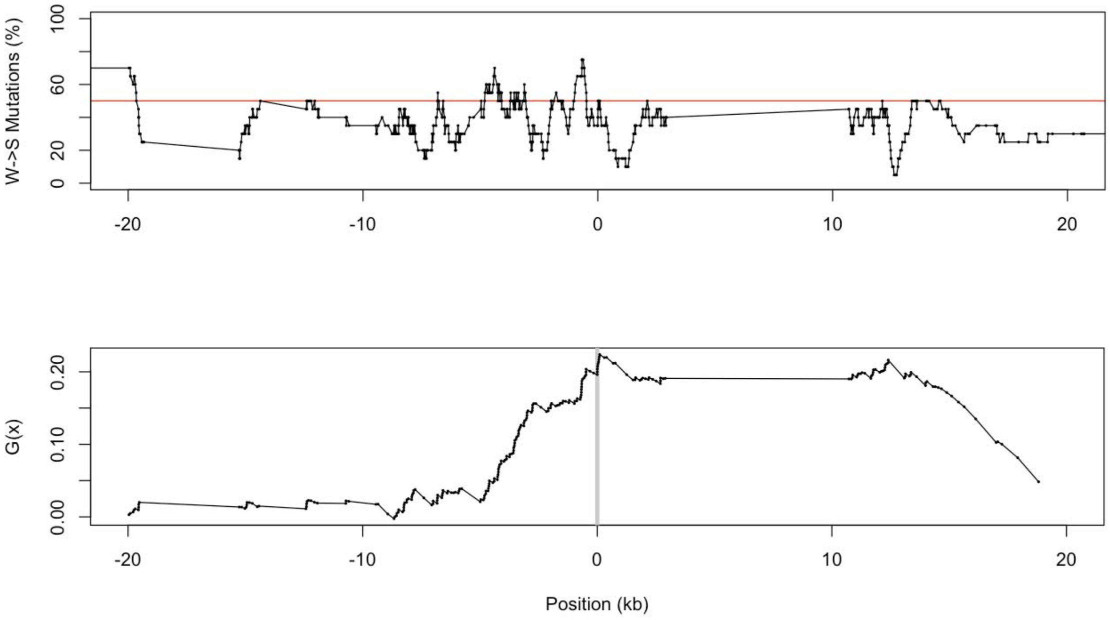

chr10:127388057-127388126 || 70 || AluJb || SINE/Alu

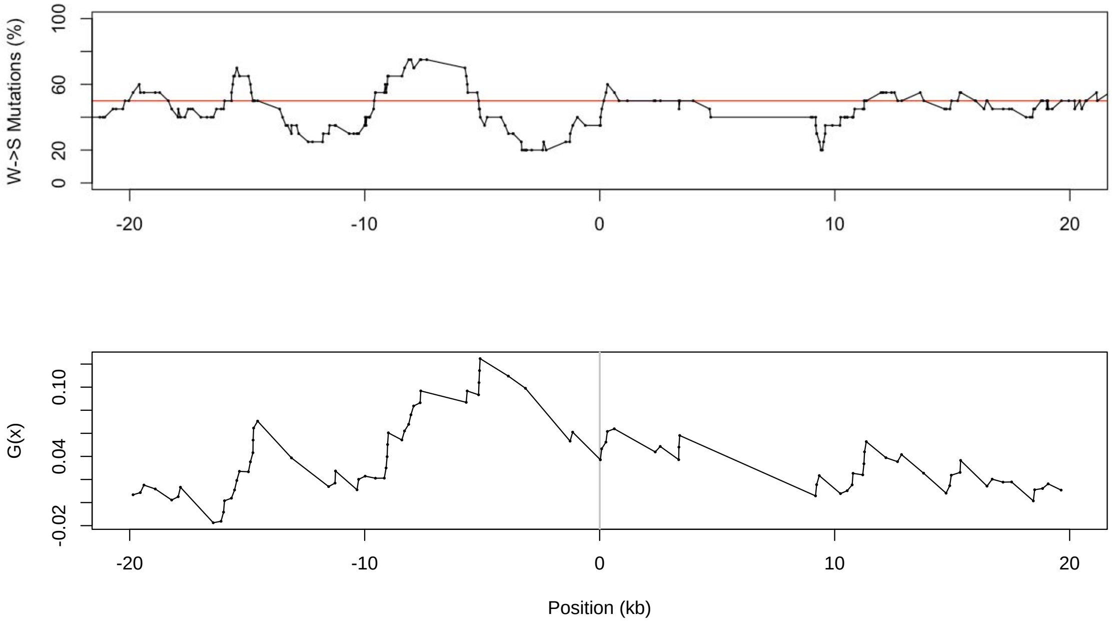

Figure S13 (cont.)

chr4:24786033-24786290 || 258 || MLT1H || LTR/ERVL-MaLR

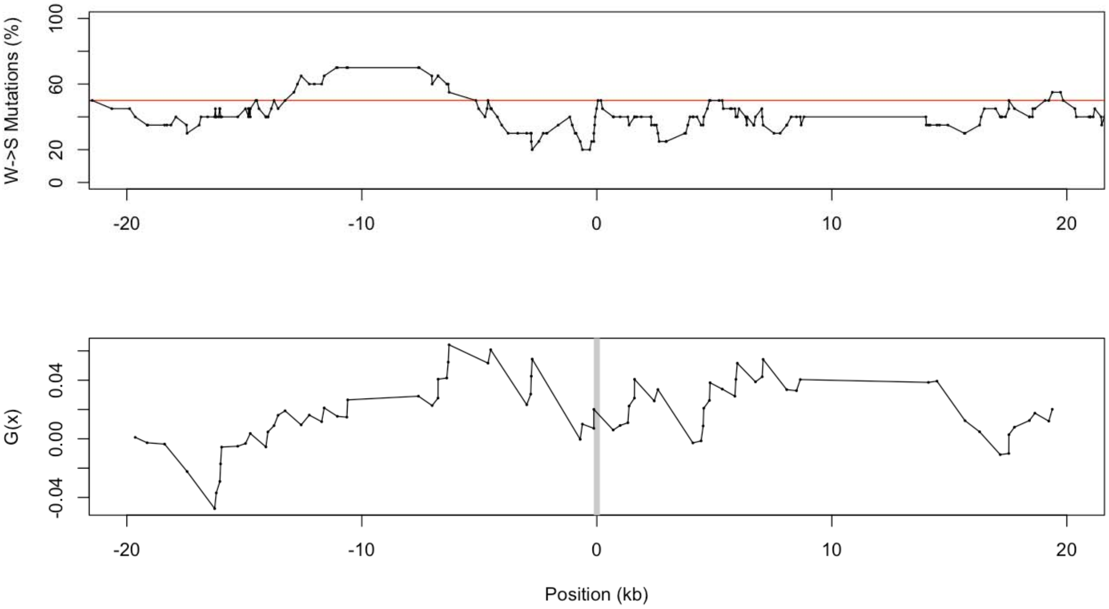

chr4:93437704-93437803 || 100 || MER103C || DNA/hAT-Charlie

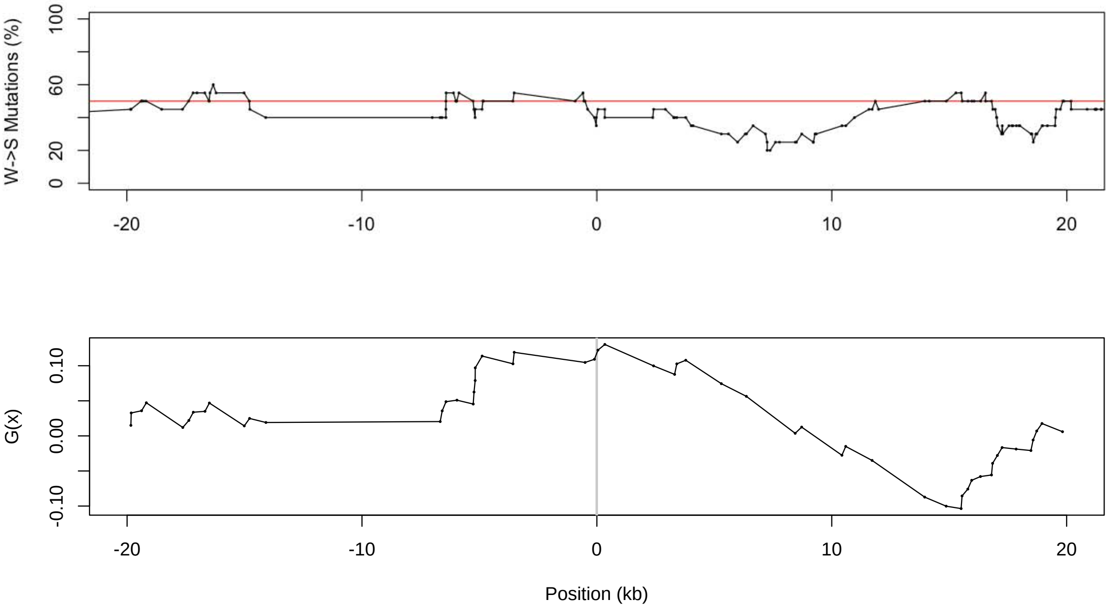

Figure S13 (cont.)

chr3:128796396-128796462 || 67 || MLT1J || LTR/ERVL-MaLR

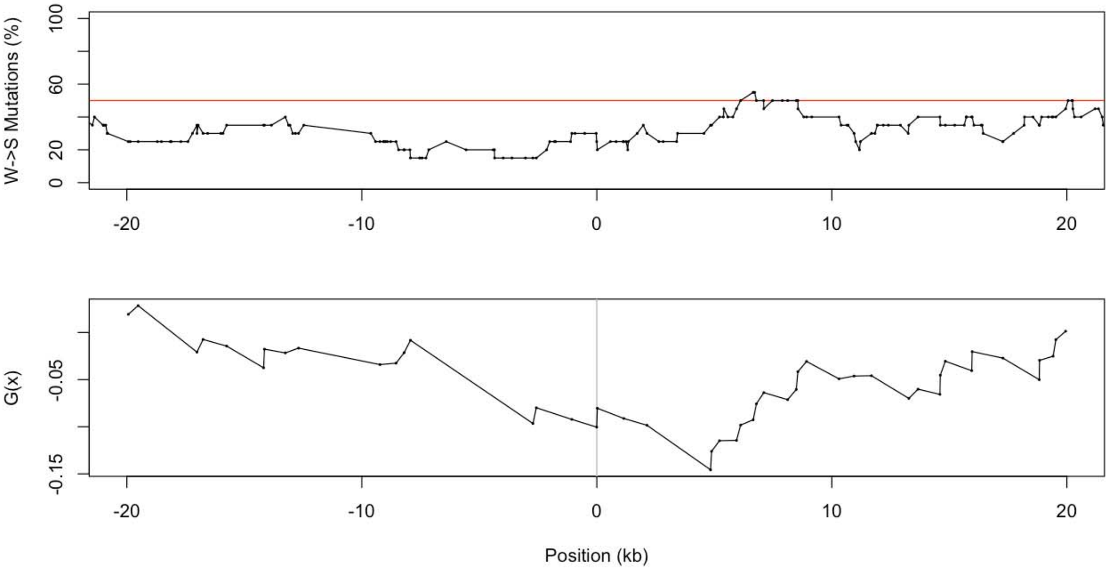

chr16:73026569-73026653 || 85 || HAL1 || LINE/L1

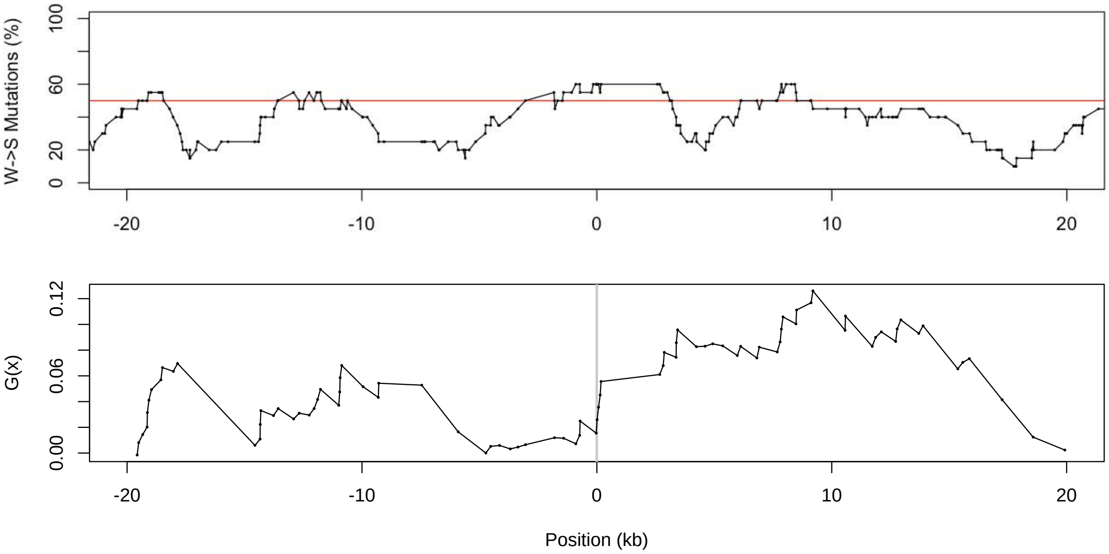

Figure S13 (cont.)

chr12:111712004-111712071 || 68 || AluJb || SINE/Alu

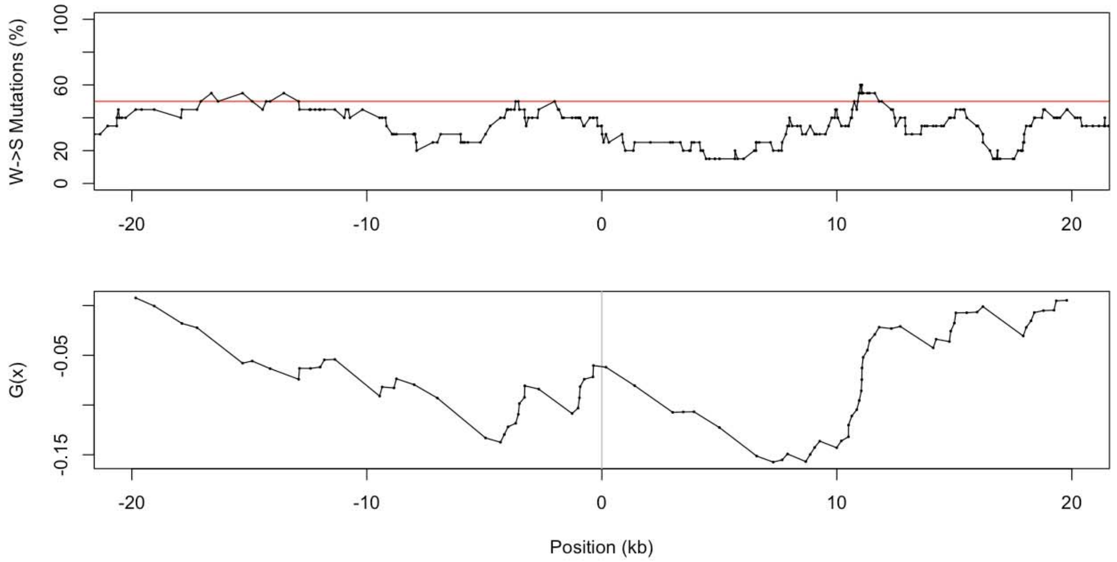

chr5:80569937-80570083 || 147 || MER5C || DNA/hAT-Charlie

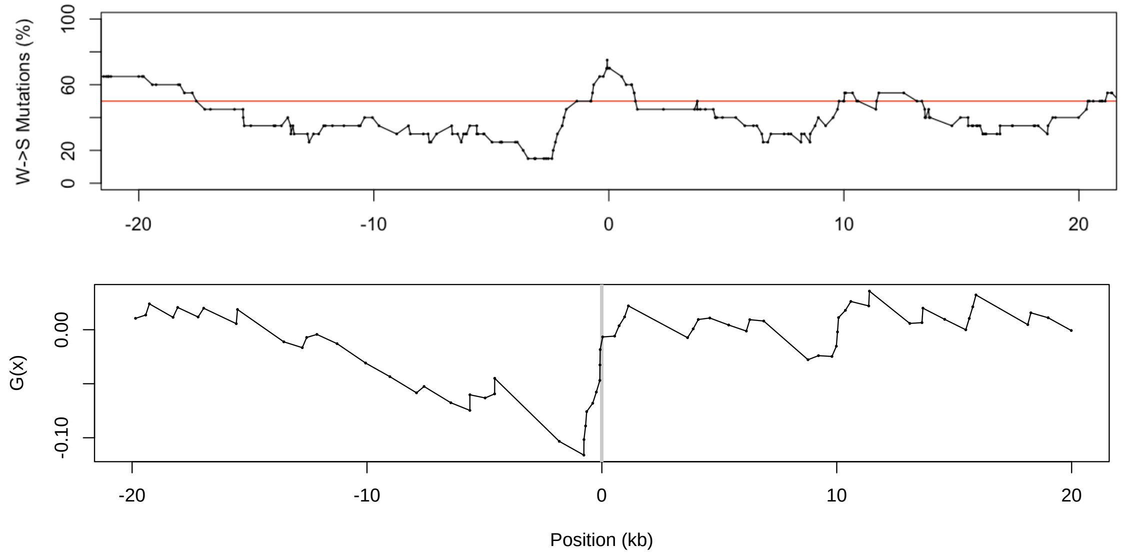

Figure S13 (cont.)

chr8:6686600-6686703 || 104 || L2a_3end || LINE/L2

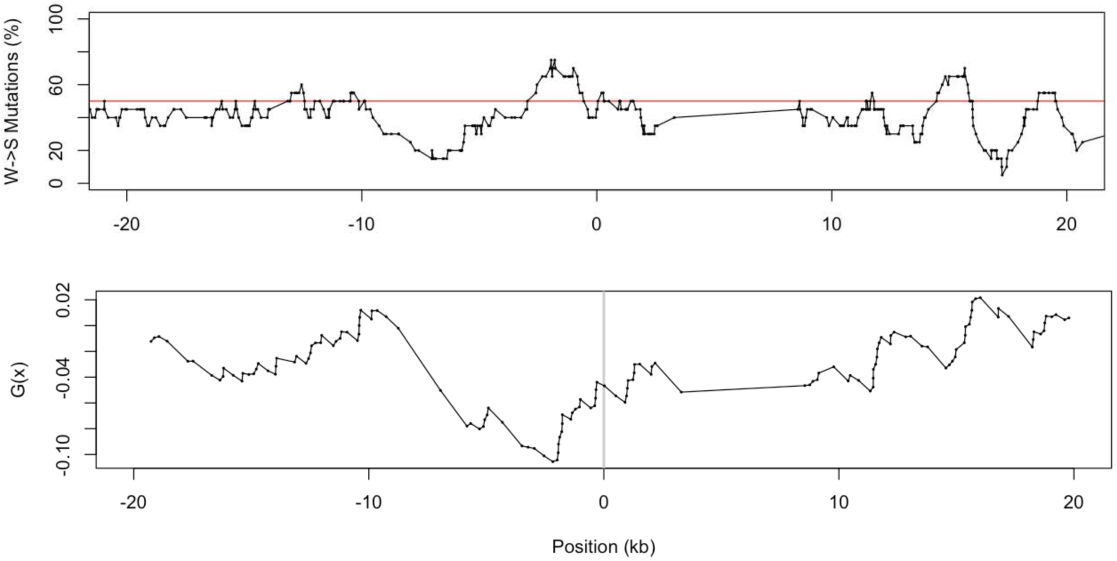

chr1:40731503-40731617 || 115 || L1M5_orf2 || LINE/L1

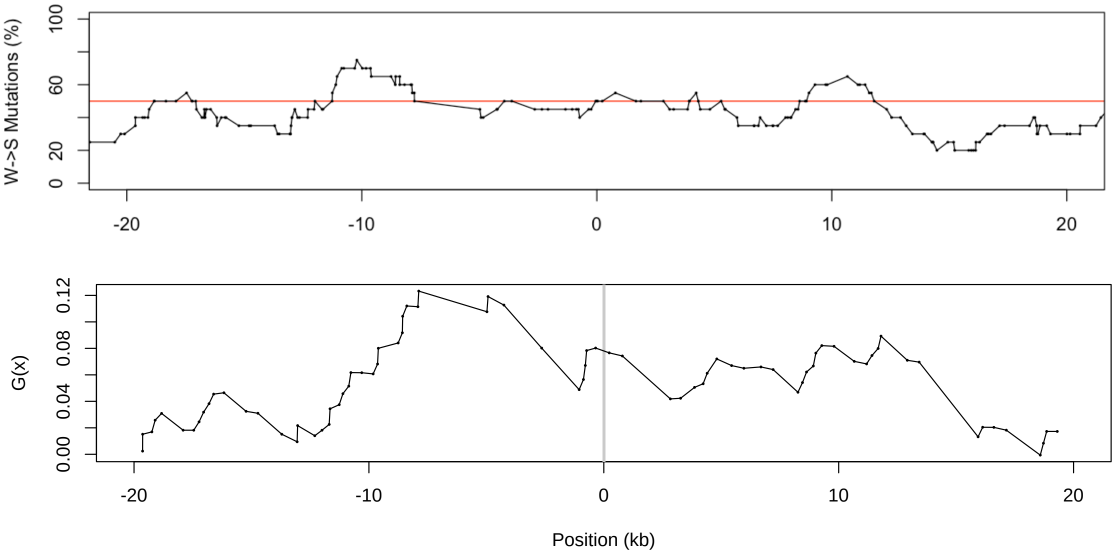

Figure S13 (cont.)

chr3:130075118-130075234 || 117 || L2a_3end || LINE/L2

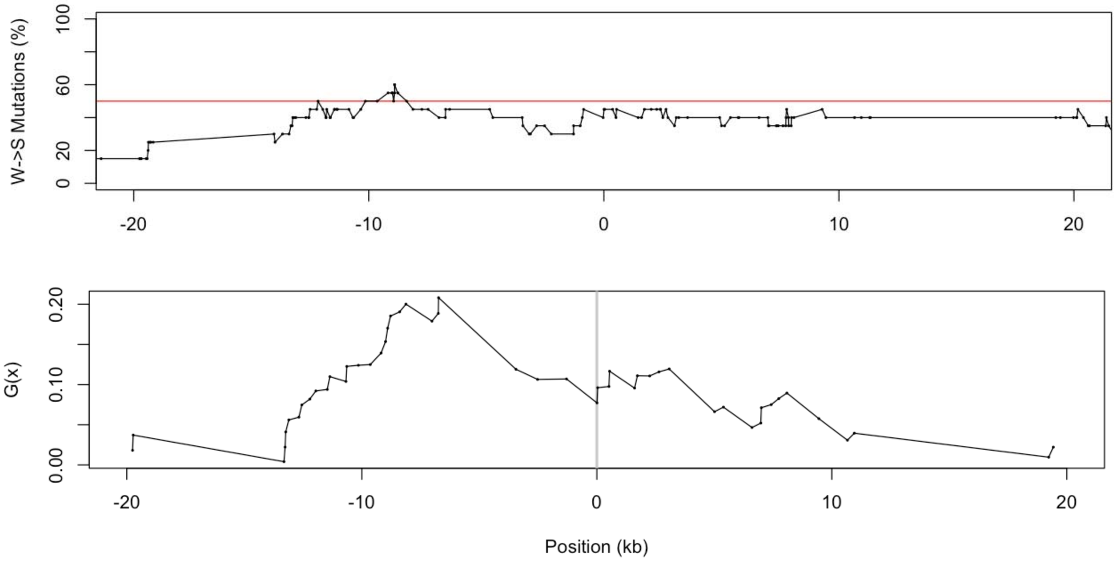

chr5:33459888-33460009 || 122 || MLT1K || LTR/ERVL-MaLR

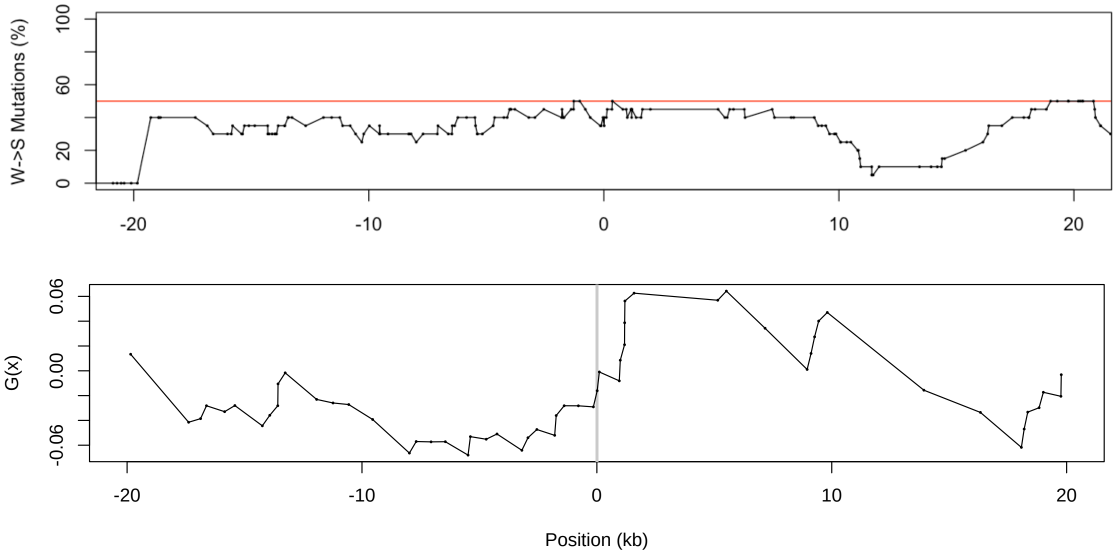

Figure S13 (cont.)

chr13:106018824-106018885 || 62 || MIRc || SINE/MIR

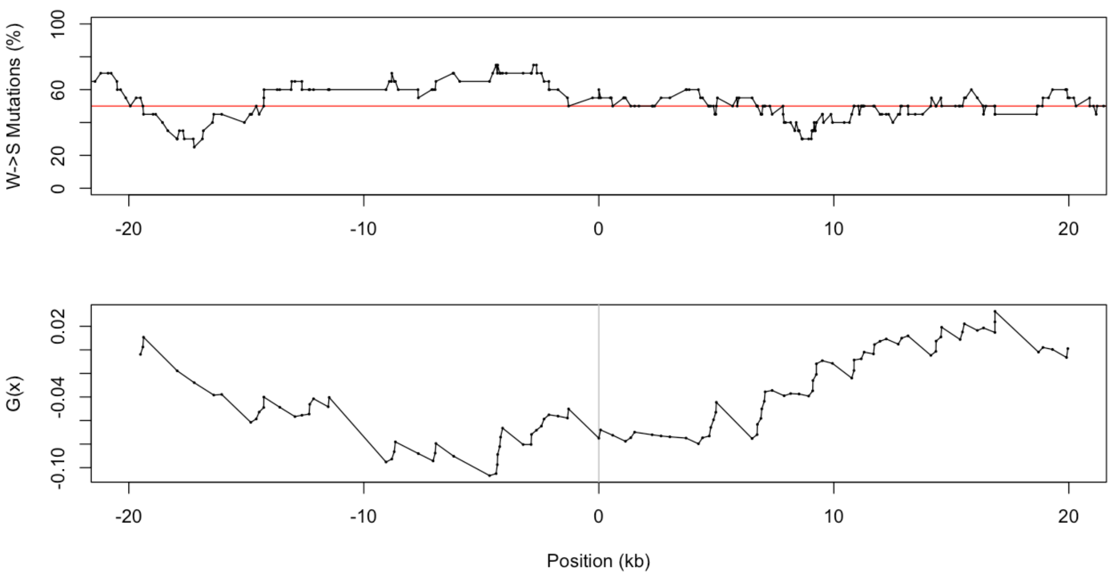

chr10:127388023-127388143 || 121 || AluJb || SINE/Alu

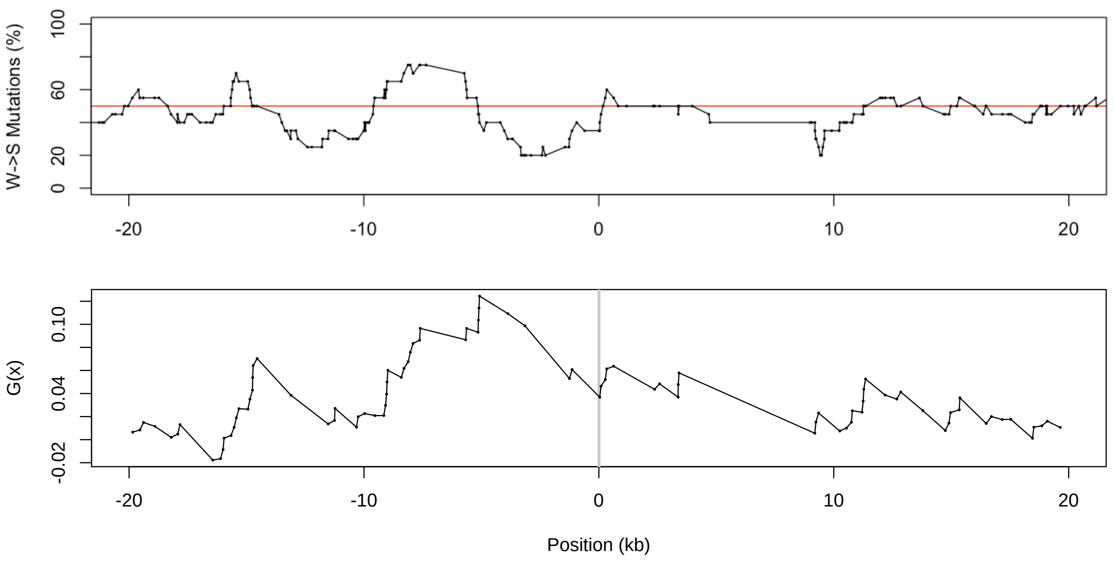

Figure S13 (cont.)

chr11:27518143-27518239 || 97 || THE1C || LTR/ERVL-MaLR

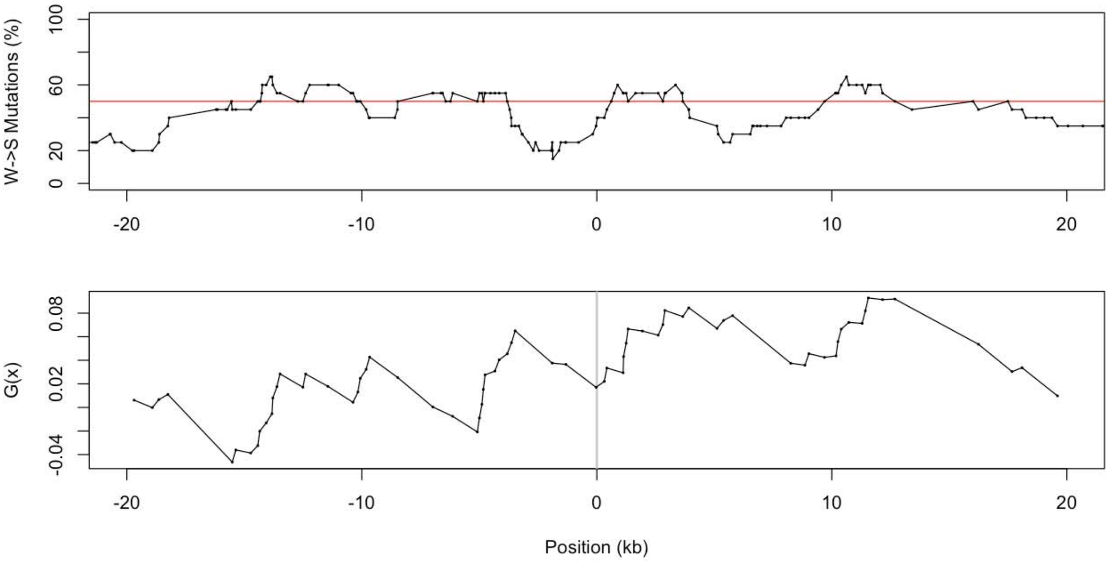

chr2:227838799-227838895 || 97 || THE1C || LTR/ERVL-MaLR

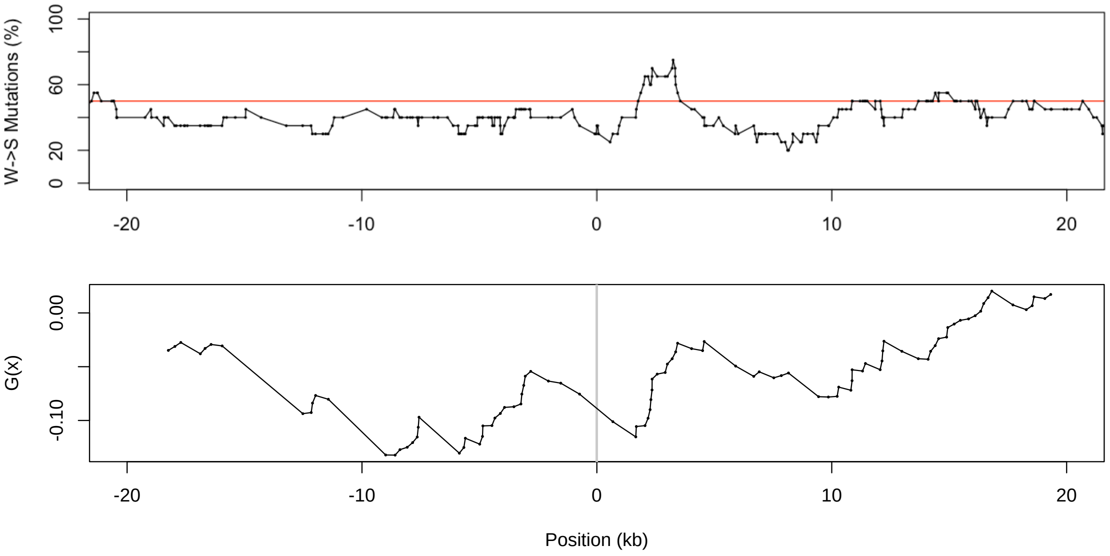

Figure S13 (cont.)

chr5:162788525-162788617 || 93 || AluJb || SINE/Alu

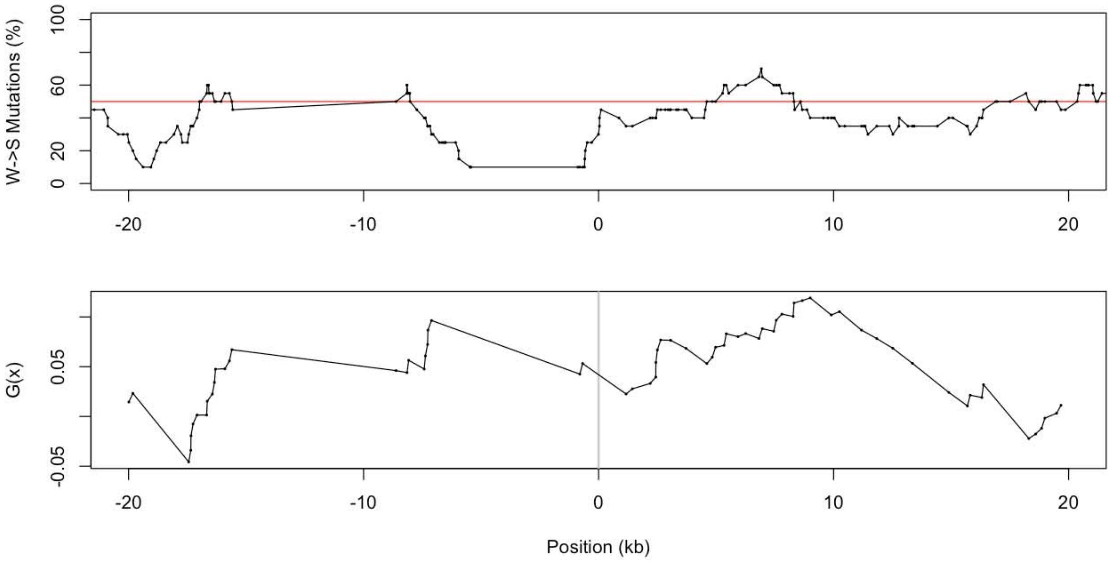

chr5:126628093-126628209 || 117 || MLT1K || LTR/ERVL-MaLR

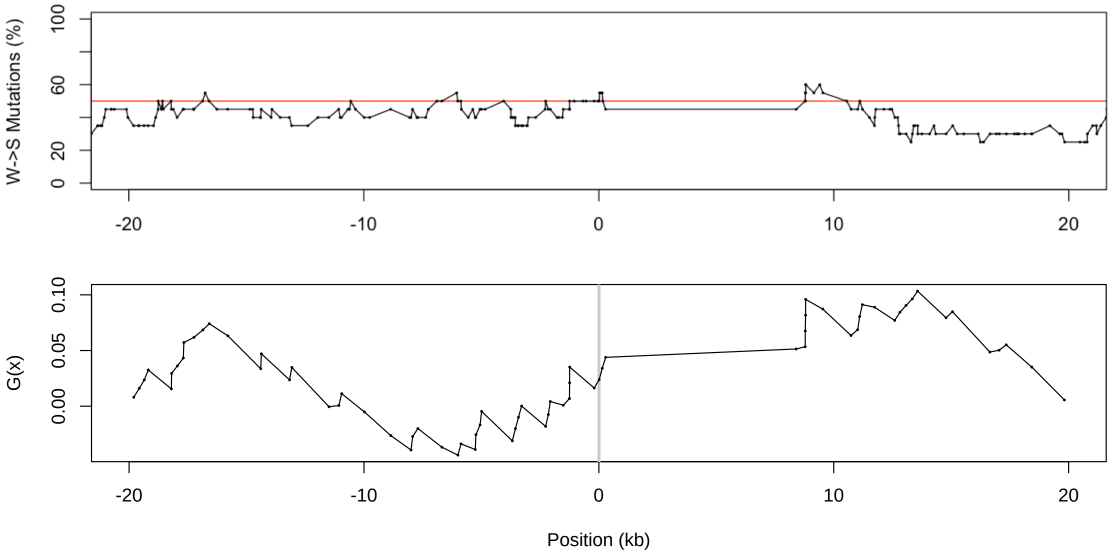

Figure S13 (cont.)

chr8:6249965-6250049 || 85 || AluJb || SINE/Alu

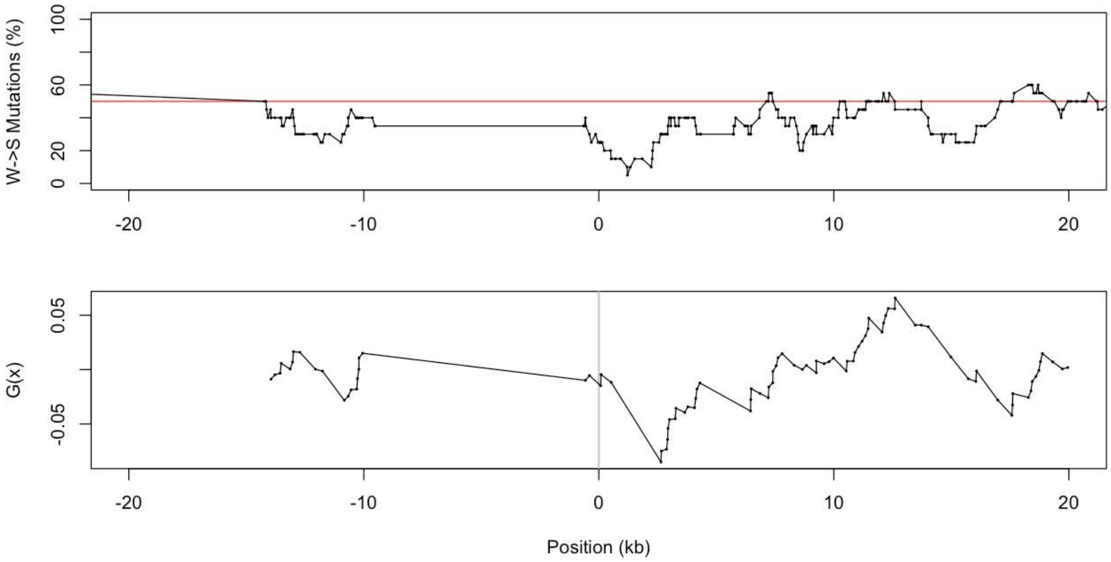

chr11:36489082-36489164 || 83 || MIR || SINE/MIR

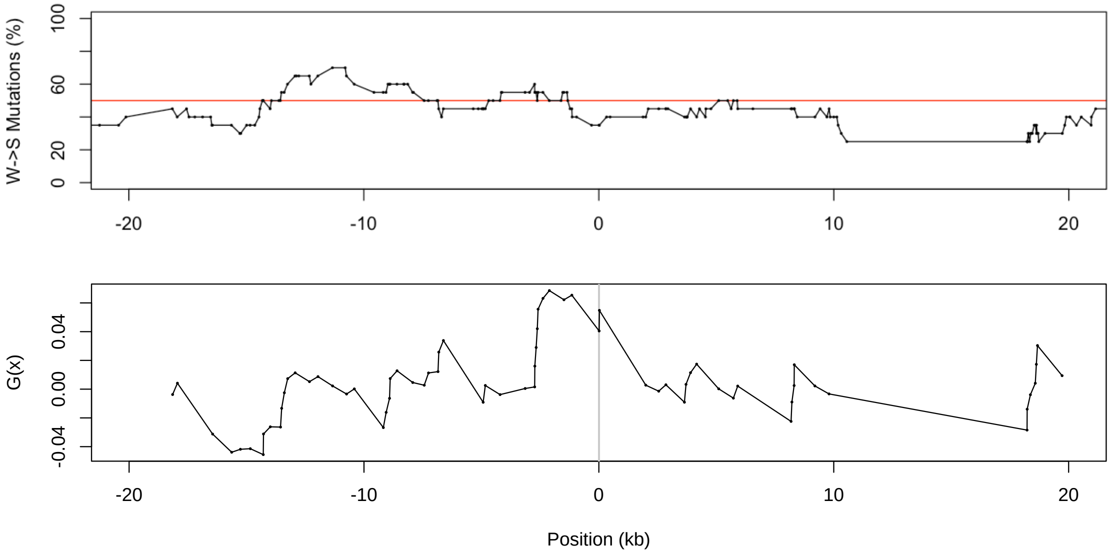

Figure S13 (cont.)

chr22:23178179-23178296 || 118 || AluSx || SINE/Alu

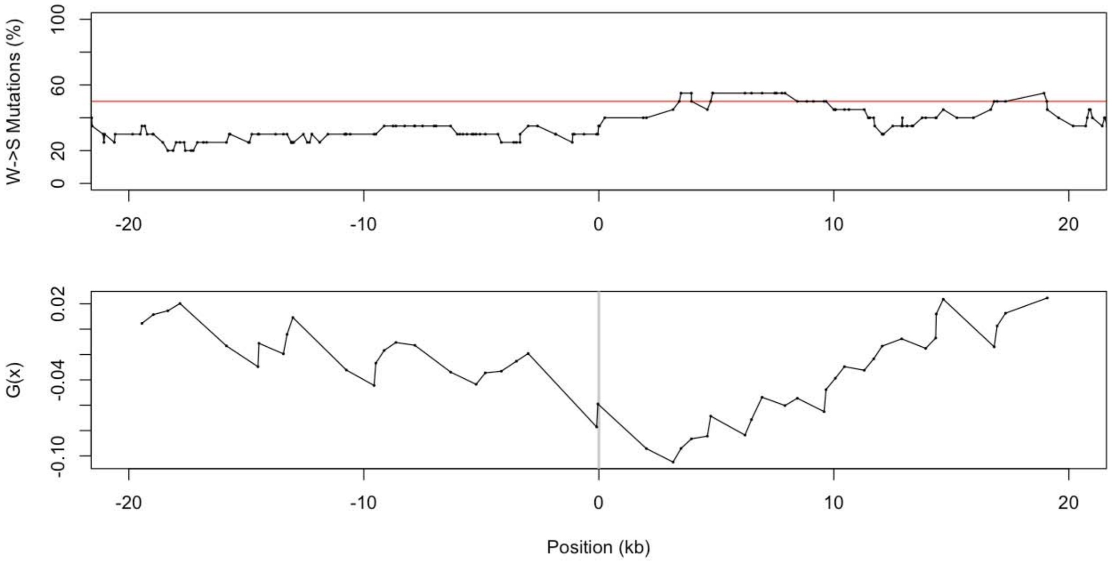

chr7:6713777-6713860 || 84 || LTR37A || LTR/ERV1

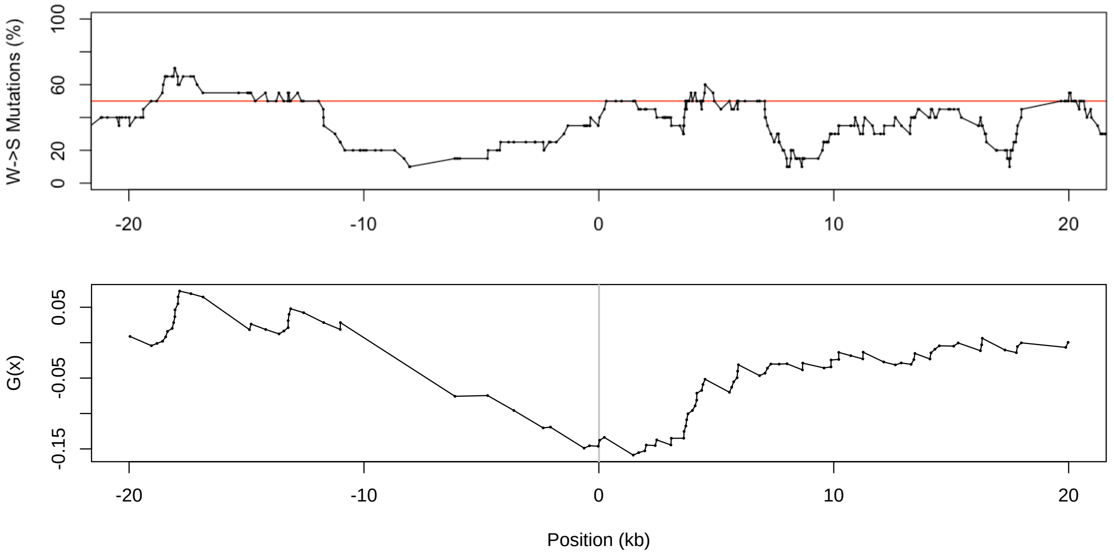

Figure S13 (cont.)

chr14:23151764-23151909 || 146 || L2 || LINE/L2

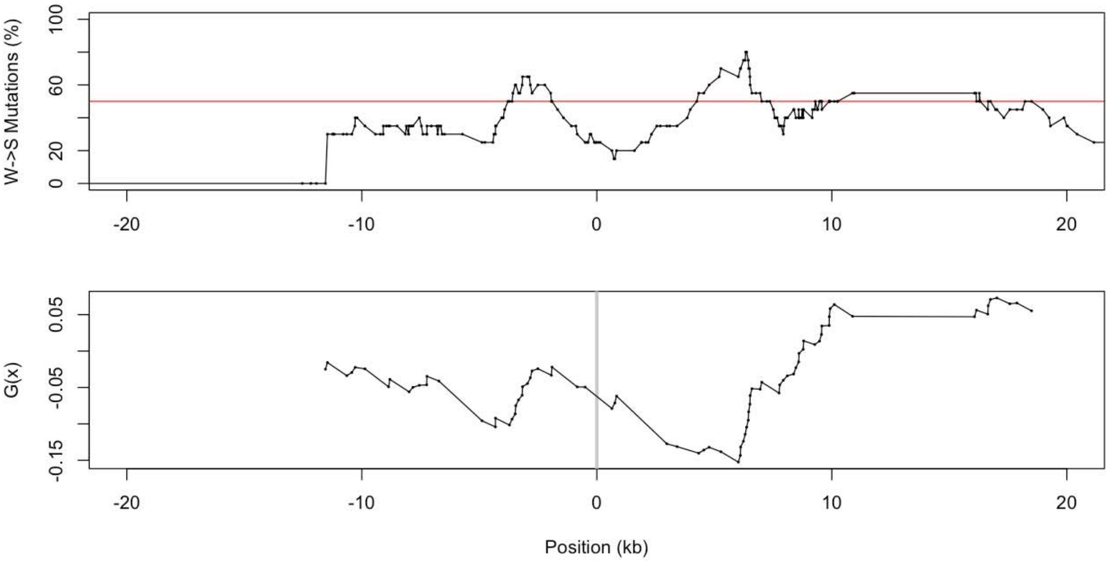

chr15:62461945-62462242 || 298 || AluSx || SINE/Alu

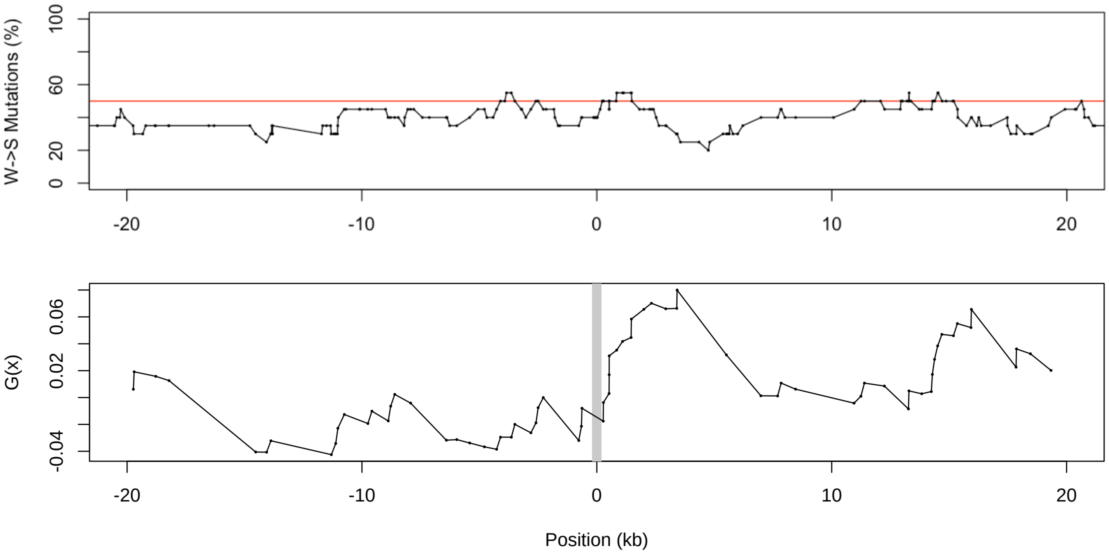

Figure S13 (cont.)

chr11:124535652-124535733 || 82 || MIR || SINE/MIR

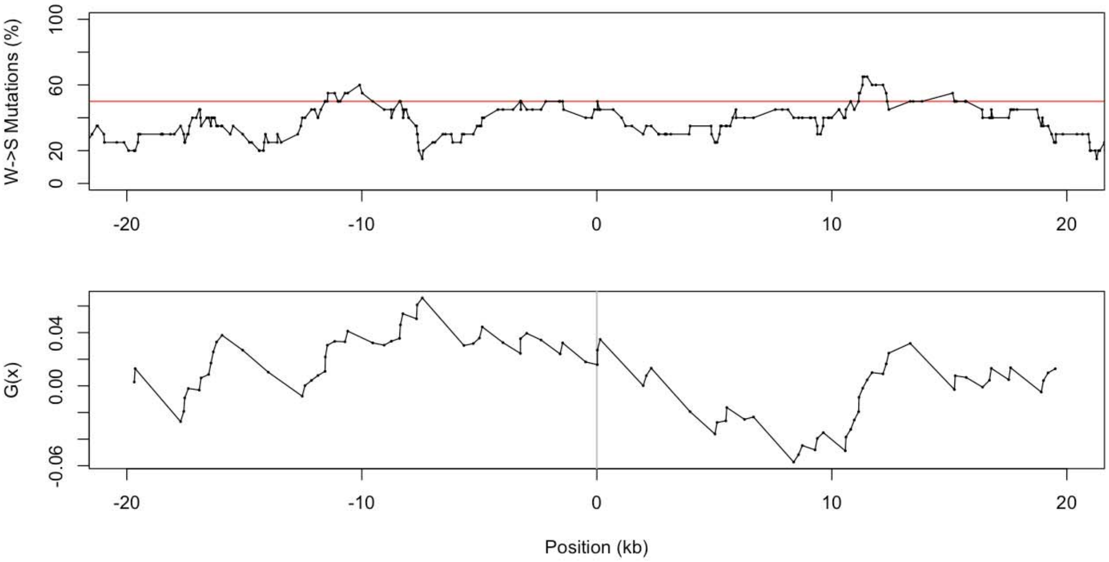

chr5:79817041-79817136 || 96 || L1MC5_3end || LINE/L1

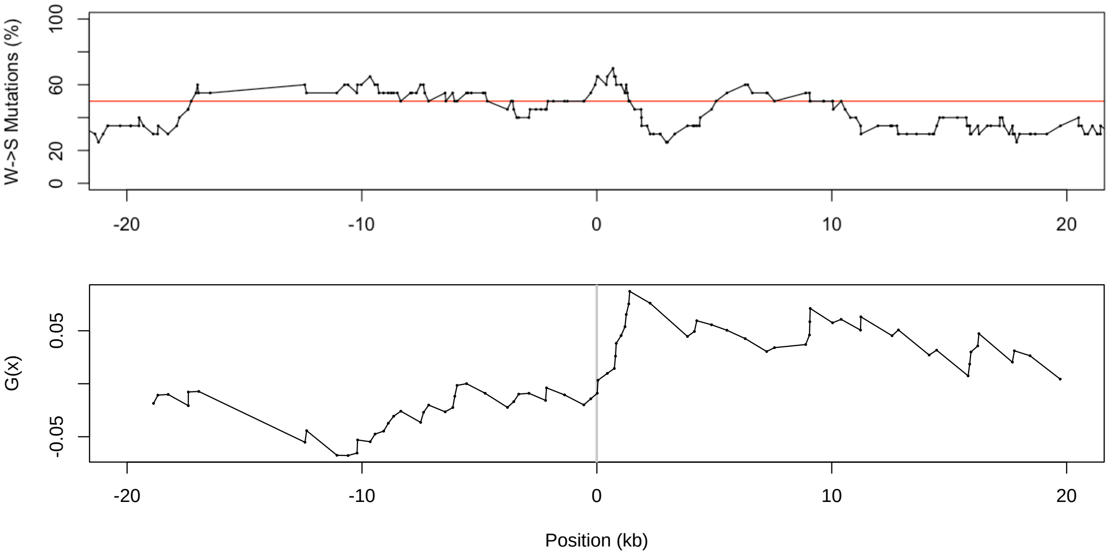

Figure S13 (cont.)

chr1:16220113-16220176 || 64 || MIRb || SINE/MIR

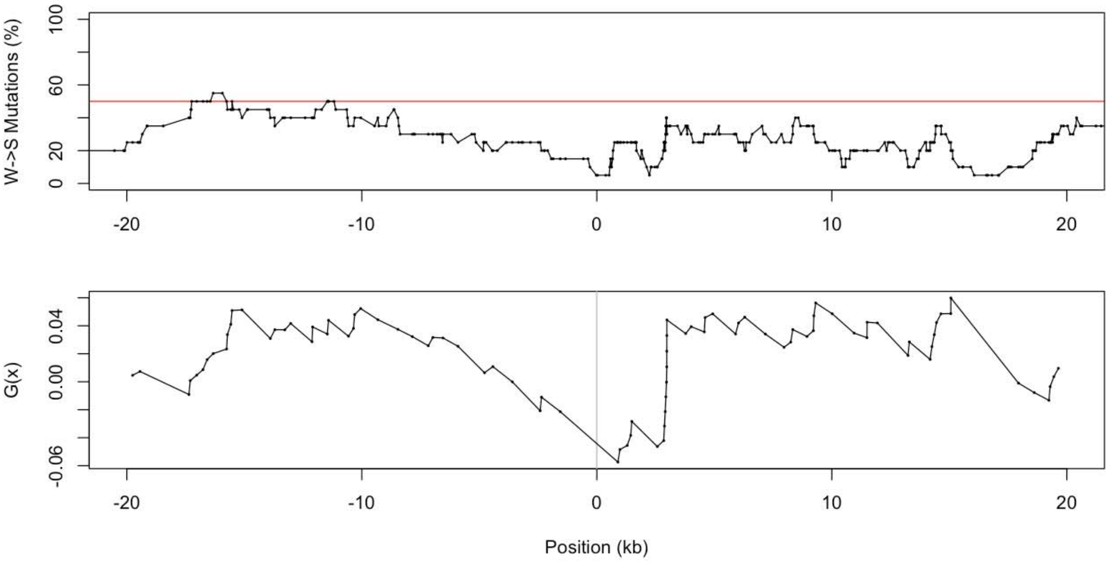

chr12:87515856-87515985 || 130 || THE1A || LTR/ERVL-MaLR

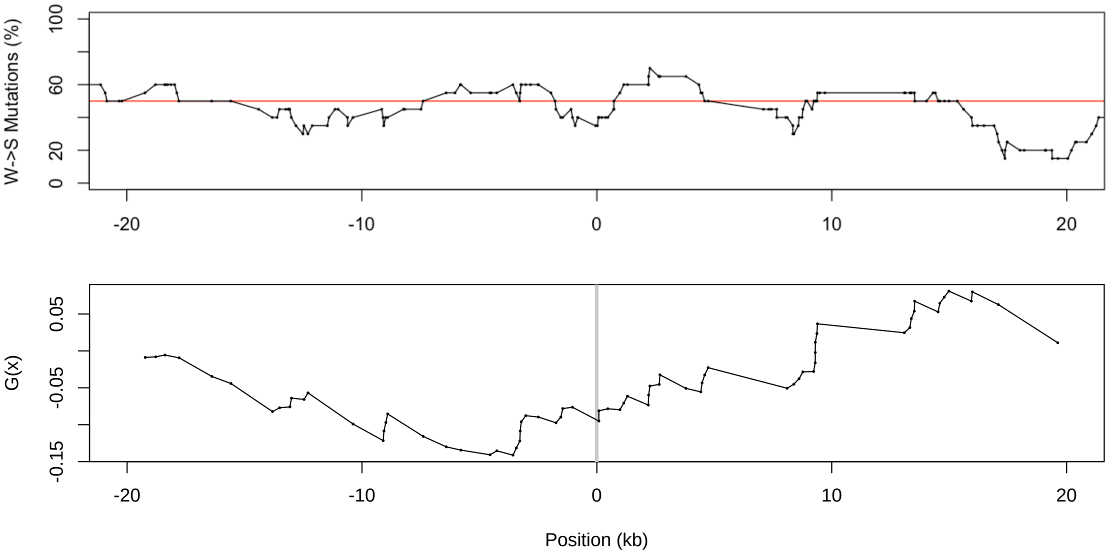

Figure S13 (cont.)

chr1:109006896-109007033 || 138 || L1MB3_3end || LINE/L1

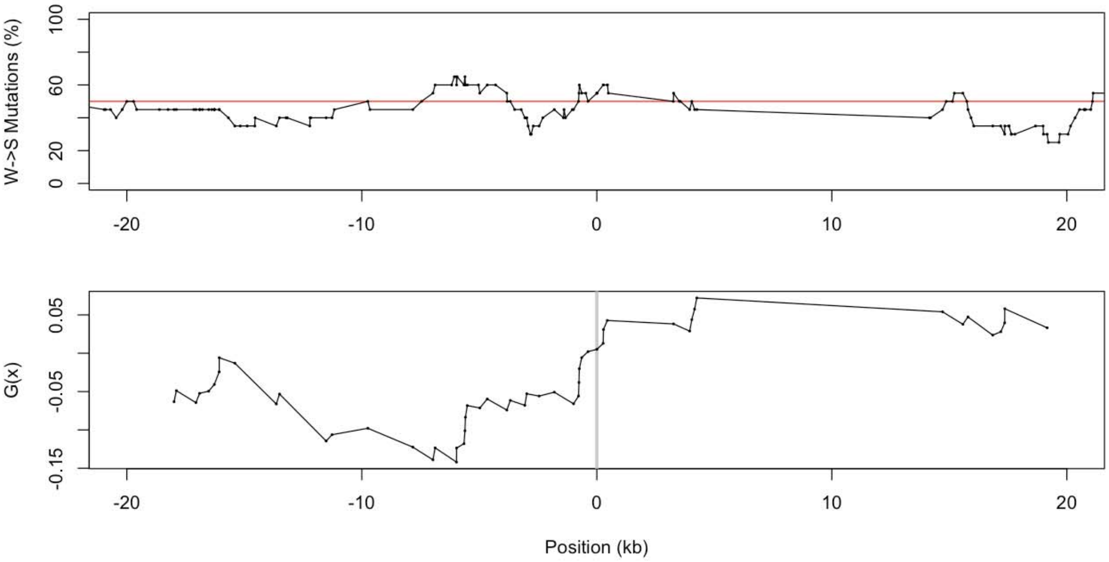

chr10:127364849-127364993 || 145 || MIRc || SINE/MIR

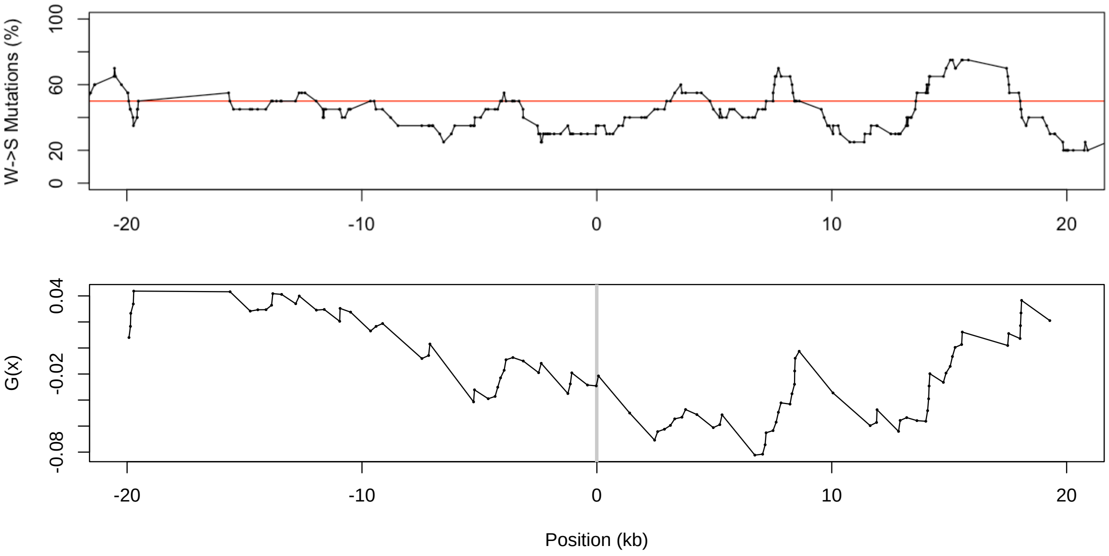

Figure S13 (cont.)

chr5:37910592-37910681 || 90 || MIR || SINE/MIR

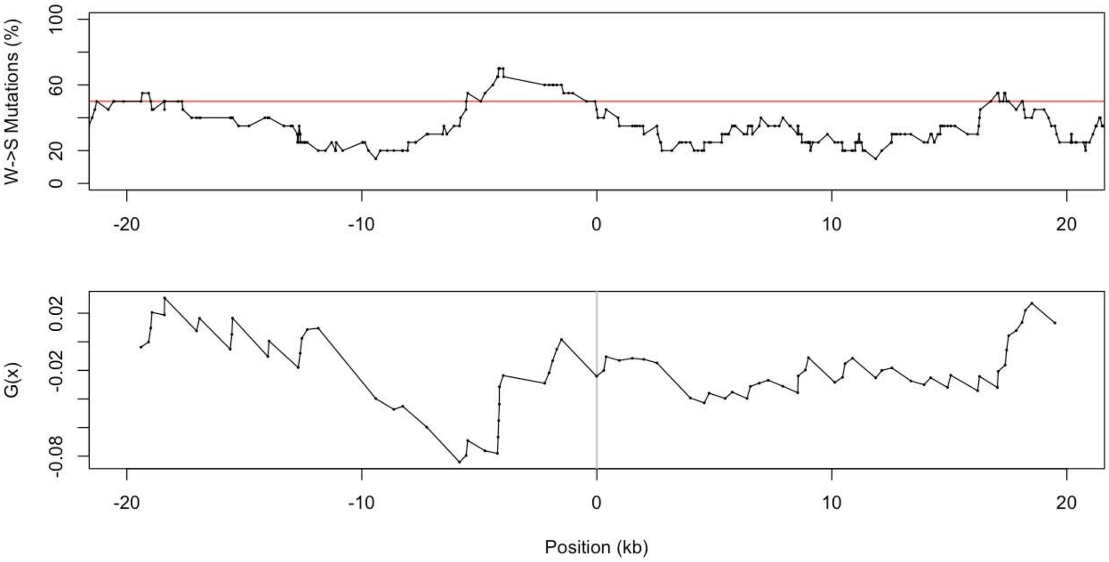

chr17:31220084-31220230 || 147 || MIRc || SINE/MIR

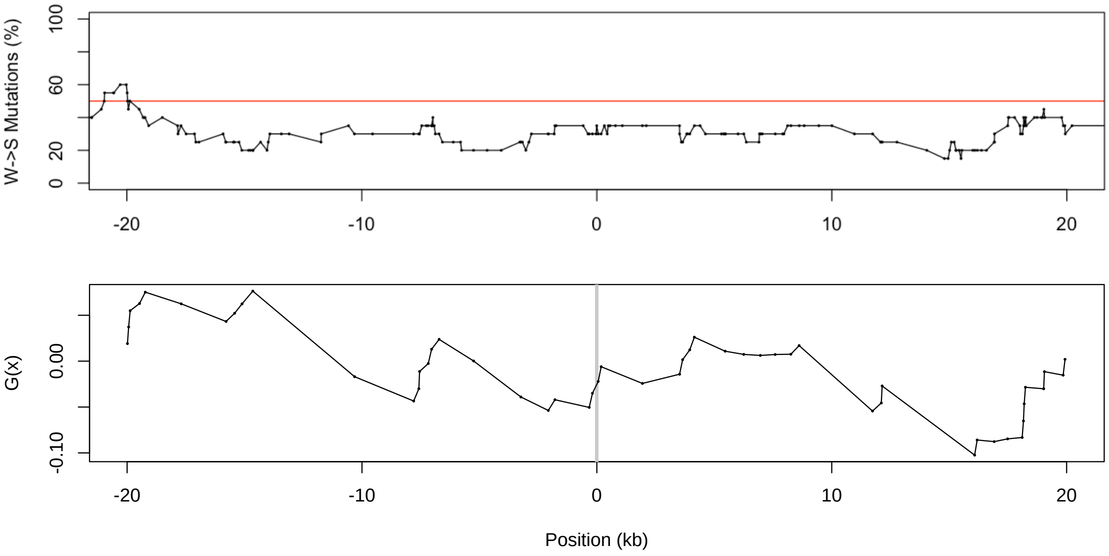

Supplement: Figure S13 — Profiles of weak-to-strong (W->S) substitution bias around 32 TE fragments found to evolve at accelerated rates in the human lineage. Two graphs are provided for each TE fragment (coordinates, size, name and class/family of each accelerated TE fragment are provided above set of graphs): i) proportion of W->S mutations computed for windows of 20 mutations (centered on each mutation). Each human-specific mutation in the shown interval is represented by a dot on the graph. The red line denotes the 50% mark; position 0 indicates the center of the accelerated TE fragment; ii) values of the G function [53] computed for W->S mutations. Hotspots of W->S mutations are highlighted by monotonically increasing G. The shaded central region of the graph corresponds to the location of the TE fragment. Examples of regions strongly affected by GC-biased gene conversion are provided in Fig. S14. (PDF) [file pone.0057323.s013.pdf]
