# Supplementary material for: Bidirectional Promoters as Important Drivers for the Emergence of Species-Specific Transcripts
Source: PLoS One. 2013 Feb 27;8(2):e57323. doi: 10.1371/journal.pone.0057323 (PMC3583895; doi:10.1371/journal.pone.0057323)
Supplement: Table S1 — Validation of BDP activity through RT-PCR of flanking genes. Within the GENCODE framework [25], PCR primers were designed to amplify transcripts from both the upstream and downstream gene loci flanking a BDP (brain, heart, kidney, liver, lung, muscle, spleen, testis). The activity of a BDP in a given tissue was validated if expected PCR product sizes were detected from both gene loci in that respective tissue. The activity of 34 BDPs was validated in at least one tissue, primer design failed in one case, and the activity of five BDPs could not be validated in any of the eight tissues tested. (PDF) [file pone.0057323.s018.pdf]

Table S1

Pair of gene loci flanking BDPs (VEGA gene IDs)	Tissues with validated BDP activity
OTTHUMG00000010130,OTTHUMG00000040465	primer design failed
OTTHUMG00000011953,OTTHUMG00000011954	brain,heart,kidney,liver,lung,muscle,spleen,testis
OTTHUMG00000011982,OTTHUMG00000011983	brain,heart,kidney,liver,lung,muscle,spleen,testis
OTTHUMG00000014158,OTTHUMG00000166289	-
OTTHUMG00000016900,OTTHUMG00000016896	brain,heart,kidney,liver,lung,muscle,spleen,testis
OTTHUMG00000017872,OTTHUMG00000166235	brain,heart,muscle,spleen
OTTHUMG00000019229,OTTHUMG00000019230	heart,testis
OTTHUMG00000019231,OTTHUMG00000019233	brain,heart,kidney,liver,lung,muscle,spleen,testis
OTTHUMG00000019985,OTTHUMG00000019983	testis
OTTHUMG00000030471,OTTHUMG00000153502	brain
OTTHUMG00000031843,OTTHUMG00000031842	brain,heart,kidney,liver,lung,muscle,spleen,testis
OTTHUMG00000033150,OTTHUMG00000034275	brain,heart,kidney,liver,lung,muscle,spleen,testis
OTTHUMG00000047996,OTTHUMG00000064974	brain,heart,kidney,liver,lung,muscle,spleen,testis
OTTHUMG00000119043,OTTHUMG00000151452	brain,heart,kidney,liver,muscle,spleen,testis
OTTHUMG00000131048,OTTHUMG00000161022	brain,heart,kidney,liver,lung,muscle,testis
OTTHUMG00000133540,OTTHUMG00000156671	-
OTTHUMG00000149816,OTTHUMG00000160764	brain,lung,spleen,testis
OTTHUMG00000153169,OTTHUMG00000130171	brain,heart,kidney,lung,muscle,spleen,testis
OTTHUMG00000154076,OTTHUMG00000133221	brain,heart,spleen,testis
OTTHUMG00000156881,OTTHUMG00000133750	kidney,testis
OTTHUMG00000160202,OTTHUMG00000160585	brain,heart,kidney,liver,lung,muscle,spleen,testis
OTTHUMG00000161789,OTTHUMG00000161785	-
OTTHUMG00000162040,OTTHUMG00000090811	brain,heart,kidney,liver,lung,muscle,spleen,testis
OTTHUMG00000162191,OTTHUMG00000096981	brain,heart,kidney,liver,lung,muscle,spleen,testis
OTTHUMG00000162277,OTTHUMG00000132428	brain,heart,liver,lung,muscle,spleen,testis
OTTHUMG00000162481,OTTHUMG00000162479	brain,heart,kidney,liver,lung,muscle,spleen,testis
OTTHUMG00000162688,OTTHUMG00000162691	brain,heart,kidney,lung,muscle,spleen,testis
OTTHUMG00000162801,OTTHUMG00000128726	-
OTTHUMG00000163513,OTTHUMG00000130304	brain,heart,kidney,liver,lung,muscle,spleen,testis
OTTHUMG00000164022,OTTHUMG00000150758	brain,heart,kidney,liver,lung,muscle,spleen,testis
OTTHUMG00000165073,OTTHUMG00000165072	brain,lung,muscle,testis
OTTHUMG00000165991,OTTHUMG00000165992	-
OTTHUMG00000166017,OTTHUMG00000166018	brain,heart,kidney,liver,lung,muscle,spleen,testis
OTTHUMG00000166065,OTTHUMG00000166066	kidney,lung
OTTHUMG00000166119,OTTHUMG00000130067	brain,heart,lung,spleen,testis
OTTHUMG00000166152,OTTHUMG00000017227	brain,heart,kidney,liver,lung,muscle,spleen,testis
OTTHUMG00000166230,OTTHUMG00000133333	brain,heart,kidney,liver,lung,muscle,spleen,testis
OTTHUMG00000166238,OTTHUMG00000020359	brain,heart,kidney,liver,lung,muscle,spleen,testis
OTTHUMG00000166252,OTTHUMG00000165993	heart,kidney,liver,lung,muscle,spleen,testis
OTTHUMG00000166301,OTTHUMG00000014589	brain,muscle,testis
