# Supplementary material for: Bidirectional Promoters as Important Drivers for the Emergence of Species-Specific Transcripts
Source: PLoS One. 2013 Feb 27;8(2):e57323. doi: 10.1371/journal.pone.0057323 (PMC3583895; doi:10.1371/journal.pone.0057323)
Supplement: Table S2 — TE fragments in PINT exons found to have accelerated rates of evolution in the human lineage. Table columns correspond to the following: A – coordinates of the TE fragment. [ ] denote a fragment that encompasses a shorter fragment (row 2), but it is part of a transcript on the reverse complement strand (included here to highlight the significance of the three human-specific mutations even in the context of a larger fragment); B – TE fragment length (bps); C – TE name; D – TE class/family; E – TE consensus coordinates corresponding to the TE fragment (parentheses indicate a match to the reverse complement strand); F – length of human-chimp-macaque ungapped alignment (bps); G – number of human-specific substitutions; H – rate of human-specific substitutions (%); I – excess of human-specific substitutions over flanking regions, computed by subtracting the rate of human-specific substitutions observed in the 4 kb flanking regions (2 kb upstream of the TE fragment, and 2 kb downstream) from the rate observed in the TE fragment (%); J – P-value associated with the excess rate (column I) estimated from a distribution of excess rates computed for specific intergenic TE homolog fragments (column L). * denotes fragments located in significant hotspots of weak-to-strong (W->S) mutations; K – P-value adjusted for multiple testing (FDR), computed in the R package with the “p.adjust(method = “BH”)” command with the P-values computed for all 51 PINT TE fragments with human-specific rates of evolution greater than 2%; L – number of specific intergenic TE homolog fragments used for significance estimate (column J). (PDF) [file pone.0057323.s019.pdf]

Table S2

A	B	C	D	E	F	G	H	I	J	K	L
chr6:32743252-32743430	179	Zaphod	DNA/hAT-Tip100	(172-356)	172	15	8.721	5.399	0*	0.0000	217
chr10:127388057-127388126	70	AluJb	SINE/Alu	35-115	70	3	4.286	3.836	0.0049	0.0538	70,275
chr4:24786033-24786290	258	MLT1H	LTR/ERVL-MaLR	229-506	257	6	2.335	1.775	0.0051	0.0538	1,763
chr4:93437704-93437803	100	MER103C	DNA/hAT-Charlie	80-180	100	3	3	2.702	0.0061	0.0538	1,643
chr3:128796396-128796462	67	MLT1J	LTR/ERVL-MaLR	338-405	67	3	4.478	4.034	0.0065	0.0538	3,845
chr16:73026569-73026653	85	HAL1	LINE/L1	(2193-2296)	85	3	3.529	2.734	0.0075	0.0538	1,207
chr12:111712004-111712071	68	AluJb	SINE/Alu	47-115	68	3	4.412	3.78	0.009	0.0538	71,892
chr5:80569937-80570083	147	MER5C	DNA/hAT-Charlie	(2-190)	147	4	2.721	2.058	0.0095*	0.0538	105
chr8:6686600-6686703	104	L2a_3end	LINE/L2	398-517	104	4	3.846	2.457	0.0095	0.0538	10,239
chr1:40731503-40731617	115	L1M5_orf2	LINE/L1	(2124-2239)	114	3	2.632	2.35	0.0129	0.0658	3,885
chr3:130075118-130075234	117	L2a_3end	LINE/L2	(398-517)	117	3	2.564	2.208	0.0165	0.0703	10,239
chr5:33459888-33460009	122	MLT1K	LTR/ERVL-MaLR	152-318	122	3	2.459	1.744	0.0171	0.0703	2,277
chr13:106018824-106018885	62	MIRc	SINE/MIR	89-152	61	2	3.279	2.892	0.0197	0.0703	26,598
[chr10:127388023-127388143]	121	AluJb	SINE/Alu	1-135	121	3	2.479	2.027	0.022	0.0703	38,601
chr11:27518143-27518239	97	THE1C	LTR/ERVL-MaLR	196-292	97	3	3.093	2.538	0.0241	0.0703	4,520
chr2:227838799-227838895	97	THE1C	LTR/ERVL-MaLR	(196-292)	97	3	3.093	2.519	0.0248	0.0703	4,520
chr5:162788525-162788617	93	AluJb	SINE/Alu	23-115	93	3	3.226	2.392	0.0252	0.0703	68,460
chr5:126628093-126628209	117	MLT1K	LTR/ERVL-MaLR	(371-489)	114	3	2.632	1.889	0.027	0.0703	3,300
chr8:6249965-6250049	85	AluJb	SINE/Alu	195-279	85	3	3.529	2.481	0.0275	0.0703	72,259
chr11:36489082-36489164	83	MIR	SINE/MIR	8-94	83	2	2.41	2.143	0.028	0.0703	17,850
chr22:23178179-23178296	118	AluSx	SINE/Alu	158-275	118	3	2.542	2.209	0.0301	0.0703	178,177
chr7:6713777-6713860	84	LTR37A	LTR/ERV1	4-94	83	2	2.41	1.958	0.0305	0.0703	131
chr14:23151764-23151909	146	L2	LINE/L2	(2591-2786)	146	3	2.055	1.475	0.0326	0.0703	11,258
chr15:62461945-62462242	298	AluSx	SINE/Alu	1-281	283	6	2.12	1.615	0.0331	0.0703	110,612
chr11:124535652-124535733	82	MIR	SINE/MIR	(21-104)	82	2	2.439	2.052	0.0373	0.0761	30,995
chr5:79817041-79817136	96	L1MC5_3end	LINE/L1	(1107-1213)	96	2	2.083	1.639	0.0421*	0.0777	1,711
chr1:16220113-16220176	64	MIRb	SINE/MIR	174-240	64	2	3.125	2.371	0.0442	0.0777	38,264
chr12:87515856-87515985	130	THE1A	LTR/ERVL-MaLR	226-355	130	3	2.308	1.678	0.0447	0.0777	1,678
chr1:109006896-109007033	138	L1MB3_3end	LINE/L1	554-676	133	3	2.256	1.575	0.0463	0.0777	2,074
chr10:127364849-127364993	145	MIRc	SINE/MIR	(27-170)	145	3	2.069	1.465	0.0466	0.0777	8,815
chr5:37910592-37910681	90	MIR	SINE/MIR	(18-110)	90	2	2.222	1.776	0.0472	0.0777	27,925
chr17:31220084-31220230	147	MIRc	SINE/MIR	85-237	140	3	2.143	1.325	0.0495	0.0789	8,445
