# Supplementary material for: Bidirectional Promoters as Important Drivers for the Emergence of Species-Specific Transcripts
Source: PLoS One. 2013 Feb 27;8(2):e57323. doi: 10.1371/journal.pone.0057323 (PMC3583895; doi:10.1371/journal.pone.0057323)
Supplement: Table S3 — Sequences cloned into pUC57 vectors for transfections into K562 cells. In addition to these three, two more sequences were created by swapping the human exon (70 bps between chr10∶127388057–127388126) for the chimp ortholog (chr10∶126680637–126680706). (PDF) [file pone.0057323.s020.pdf]

Table S3

Species	Assembly	Chromosome	Start	End	Strand
Human	hg18	10	127,387,975	127,388,208	-
Chimp	panTro2	10	126,680,555	126,680,788	-
Macaque	rheMac2	9	125,292,291	125,292,524	-
