# Supplementary material for: Bidirectional Promoters as Important Drivers for the Emergence of Species-Specific Transcripts
Source: PLoS One. 2013 Feb 27;8(2):e57323. doi: 10.1371/journal.pone.0057323 (PMC3583895; doi:10.1371/journal.pone.0057323)
Supplement: Table S4 — SplicePort scores for the SSs of the AluSx-derived exon (hg18 coordinates chr22∶23178179–23178296) in the noncoding minor C22orf45 isoform. The lack of consensus dinucleotides in macaque indicates that the splice sites were acquired in the hominoid lineage, but their splicing efficiency was diminished by three human-specific mutations. (PDF) [file pone.0057323.s021.pdf]

Table S4

Species	Acceptor (3') SS Score	Donor (5') SS Score
Human	-1.02124	-0.270026
Chimp	-0.715747	0.0283543
Macaque	N/A	N/A
