# Supplementary material for: Bidirectional Promoters as Important Drivers for the Emergence of Species-Specific Transcripts
Source: PLoS One. 2013 Feb 27;8(2):e57323. doi: 10.1371/journal.pone.0057323 (PMC3583895; doi:10.1371/journal.pone.0057323)
Supplement: Methods S1 — (DOCX) [file pone.0057323.s022.docx]

**Bidirectional promoters as important drivers for the emergence of species-specific transcripts**

Valer Gotea, Hanna M. Petrykowska, Laura Elnitski

**Supplementary Materials**

**Materials and Methods**

**GO analysis.** To find whether loci of anchors are functionally biased, we evaluated the enrichment of GO categories at these loci. To assign GO categories to anchor loci, we pooled all GO categories for all transcripts that make up all the protein-coding loci. GO assignments were obtained through NCBI gene2go and gene2refSeq annotation files (available at ftp://ftp.ncbi.nih.gov/gene/DATA/), and kgXref.txt cross-reference file provided with the UCSC hg18 annotation database. For every GO category associated with the anchor loci we performed a hypergeometric test to find significant enrichment over a background comprising all potential protein-coding anchor loci. The Bonferonni correction was used to account for multiple testing.

**Minigene splicing assay.** Recombinant clones with human, chimp and macaque sequences were transiently transfected into K562 cells. The sequences of interest (Table S4) were artificially synthesized by GeneWiz (South Plainfield, NJ), after which they were cloned into pUC57 plasmids. Subsequent cloning transferred the inserts to a mini-gene splicing vector. Initially, a 10 μl recombination reaction (BP) contained 150 ng of the attB containing pUC57 clones mixed with 200 ng of pDONR vector (Invitrogen). Then, 2 μl of 5-fold BP clonase reaction buffer mixture was added. TE buffer, pH 8, was used to complete the reaction volume. The reaction was incubated at 25°C for 1 h. To inactivate the enzyme, 1 μl of Proteinase K (2 mg/ml) solution was added to the reaction, which was further incubated at 37°C for 10 min. One Shot TOP10 chemically competent *E. coli* cells from Invitrogen (catalog number C 404003) were then used for the transformation step. The transformed cells were plated on kanamycin-supplemented LB plates pre-warmed at 37°C for 30 min. Single colonies were inoculated in 5 ml of LB medium with 50 μg/ml kanamycin and plasmid DNA was isolated. To construct custom transfection vectors, we performed 10 μl ‘LR’ recombination reactions that were set up similarly to the above-described BP recombination. In brief, 50-150 ng of the pDONR clones were mixed with 150 ng of pDESTsplice vector [1]. Then, 2 μl of LR Clonase II enzyme mix was diluted five-fold from its commercially supplied concentration. The additional volume was made up with TE buffer, pH 8.0. The reaction was incubated at 25°C for 1 h. The reaction was treated with 1 μl of Proteinase K (2 mg/ml) for 10 minutes at 37°C, transformed and plated on carbenicillin-supplemented LB-Agar plates (100 μg/ml) and screened for positive clones. In this destination vector, sequences are inserted between constitutively expressed exons from the rat insulin 2 gene [1]. The positive clones were confirmed by digestion with BsrGI and DNA sequencing by MCLAB (South San Francisco, CA). For the transfection step, we used 4x10^5^ K562 cells, which were transfected with 0.4 μg of DNA (in triplicates) by electroporation (using the Lonza 96-well nucleofector II) in 12 1-ml well plates. After 24 hours, 3 wells were combined to harvest RNA using the RNeasy® mini kit (Qiagen) and reverse transcription was performed using the iScript™ cDNA synthesis kit (Bio-Rad Laboratories). To generate cDNA, 900 ng of the RNA was used in a 20 μl iScript reaction mix and incubated in a PCR machine under the following conditions: 5 minutes at 25*°*C, 30 minutes at 42*°*C and 5 minutes at 85*°*C. The resulting cDNA was amplified by PCR using primers specific to the rat insulin 2 exons flanking the inserted genomic regions
(5’-CCTGCTCATCCTCTGGGAGC-3’, 5’-AGGTCTGAAGGTCACGGGCC-3’). Each PCR reaction contained 1 μl cDNA template, 5 μl 10x PCR buffer (15 mM MgCl_2_, Applied Biosystems), 0.5 μl (2.5U) AmpliTaq® DNA polymerase (Applied Biosystems), 1 μl dNTP, 1 μl of each of the two primers (12.5 μM), 40.5 μl H_2_O. Conditions were set at 95°C denaturation for 5 min, followed by 35 cycles of 95°C for 1 min, 62°C for 1 min, and 72°C for 1 min, with a final 72°C hold for 5 min. The amplified cDNA was visualized by gel electrophoresis on a 2% agarose gel. Quantitation of the cDNA products was performed using PCR products amplified with primers similar to the ones described above, but to which a tag with 6-carboxyfluorescein was added at the 5’ end to enable the analysis of their size and quantity. The samples were run on the Applied Biosystems (ABI) Prism 3100 Genetic Analyzer, which included the use of a 36-cm capillary array and POP4 polymer. All samples were run with the ABI internal lane size standard ROX 500, which can be used to size fragments between 35-500 bps. The samples were analyzed using ABI GeneScan (Version 3.7 II) and Genotyper (Version 2.5) software.

**Identifying hotspots of weak-to-strong (W->S) mutations.** To identify regions likely to evolve under the influence of GC-biased gene conversion, we analyzed the profile of
W->S mutation across a 40 kb region centered on the TE fragment of interest. We plotted both the proportion of W->S mutations in sliding windows of 20 human-specific mutations [2], as well as the *G*(*x*_k_) function [3] constructed using only W->S mutations (alignment gaps were used to adjust the total region length and position of mutation for the purpose of this test). To determine if each fragment was located in a significant hot spot of W->S mutations, we first determined the characteristics of the hot spot (Δ*G*, number of consecutive segments with decreasing *G* values) where the case. We then estimated the probability of observing a hot spot with these characteristics by Monte Carlo simulations (100,000 replicates). Given the number of W->S mutations and the total length of the region (adjusted for gaps), we randomly sampled (without replacement) positions in this space. We then computed values of the *G* function for each mutation position, and searched for a hotspot with equal or higher Δ*G* values. The number of replicates in which such a hotspot was found was used to determine the significance of the hotspot in which the TE fragment of interest resides.

**Testing the influence of promoter regions on the emergence of novel transcripts.** To quantify the evolutionary importance of active promoter regions for the emergence of novel transcripts, we compared the number of PINTs found in this study with the number of potential novel transcripts that can be found in regions further away from active promoters. For this purpose we defined the search space to be the equivalent of all 1-kb regions located upstream of protein-coding transcripts that are conserved in mouse. In order to provide a conservative estimate for the importance of promoter regions, we maximized the number of potential novel transcripts. Our original set included transcripts in the spliced EST annotation track that did not overlap protein-coding regions, as well as all RefSeq and UCSC knownGene transcripts that lack annotated ORFs. From these we selected those transcripts for which we could not map their first exon to the mm9 mouse genome assembly (using the UCSC liftOver tool with the –minMatch=0.1 setting), or those for which we could map the first exon to the mouse genome but for which we could not find a mouse transcript with the annotated TSS within 250 bps from the mapped TSS of the human transcript. We also maximized the search space for potential novel transcripts by considering all 1 kb regions upstream of protein coding transcripts as potentially hosting PINTs. By overlapping all these upstream regions, we obtained a set of 19,472 genomic regions with sizes within 1 – 3.2 kb range. We then randomly sampled the same number of regions (19,472) from the human genome, while maintaining with the exact same size and chromosomal distribution as the original set. We did not allow regions to overlap the original set or regions with missing sequence (N-blocks) by more than 250 bps. We then counted the number of regions in which we found the TSS of a potential novel transcript, and repeated the procedure 10,000 times to generate a distribution of expected regions where novel transcripts could emerge (Figure S16). No condition on the strand of potential novel transcripts was imposed as it was the case for PINTs, and therefore the expected number of regions with novel transcripts likely represents an overestimate, making the comparison a conservative one.

**Supplementary File**

Details about PINT and anchor transcripts are provided as a supplementary file (PINTs_anchors_details.txt). The file contains 1,068 lines, with a first header line and 1,067 lines with details of 1,067 PINT-anchor pairs in a tab delimited format (7 columns): PINT accession number, PINT coordinates (chr:start-end), PINT strand, size of BDP (bps), anchor strand, anchor coordinates, anchor accession number. All coordinates and strands correspond to the hg18 UCSC human genome assembly.

**Supplementary References**

1. Kishore S, Khanna A, Stamm S (2008) Rapid generation of splicing reporters with pSpliceExpress. Gene 427: 104-110.

2. Ratnakumar A, Mousset S, Glemin S, Berglund J, Galtier N, et al. (2010) Detecting positive selection within genomes: the problem of biased gene conversion. Philos Trans R Soc Lond B Biol Sci 365: 2571-2580.

3. Tang H, Lewontin RC (1999) Locating regions of differential variability in DNA and protein sequences. Genetics 153: 485-495.
